# Genome-Based Classification and Therapy of Prostate Cancer

**DOI:** 10.3390/diagnostics8030062

**Published:** 2018-09-02

**Authors:** Arlou Kristina Angeles, Simone Bauer, Leonie Ratz, Sabine M. Klauck, Holger Sültmann

**Affiliations:** Division of Cancer Genome Research, German Cancer Research Center (DKFZ), German Cancer Consortium (DKTK), and National Center for Tumor Diseases (NCT), Im Neuenheimer Feld 460, Heidelberg D-69120, Germany; a.angeles@dkfz.de (A.K.A.); simone.bauer@dkfz.de (S.B.); ratzleonie@hotmail.com (L.R.); s.klauck@dkfz.de (S.M.K.)

**Keywords:** prostate cancer, genomics, copy number alterations, mutations, molecular markers, precision medicine, patient stratification

## Abstract

In the past decade, multi-national and multi-center efforts were launched to sequence prostate cancer genomes, transcriptomes, and epigenomes with the aim of discovering the molecular underpinnings of tumorigenesis, cancer progression, and therapy resistance. Multiple biological markers and pathways have been discovered to be tumor drivers, and a molecular classification of prostate cancer is emerging. Here, we highlight crucial findings of these genome-sequencing projects in localized and advanced disease. We recapitulate the utility and limitations of current clinical practices to diagnosis, prognosis, and therapy, and we provide examples of insights generated by the molecular profiling of tumors. Novel treatment concepts based on these molecular alterations are currently being addressed in clinical trials and will lead to an enhanced implementation of precision medicine strategies.

## 1. Introduction

With almost 1 million new cases per year, prostate cancer (PCa) is the most prevalent malignancy in men worldwide and causes about 300,000 deaths annually [1]. Age, ethnicity, and family history are established risk factors for the disease. Age is by far the most important one: Only 30% of all cases are diagnosed under the age of 65 years in the U.S. [2]**,** and the 10-year risk to develop PCa for men aged 30, 60, and 70 years is 0.01%, 4.8%, and 5.5%, respectively [2]. Lifestyle and environmental risk factors are also implicated in PCa, as evidenced by epidemiological data gathered in Western countries, in which the disease is more prevalent compared to other regions of the world, including data from migrant populations [3]. The higher incidence in African Americans compared to Asian Americans points to the existence of PCa-associated genetic risk factors [4]. Indeed, at least 100 common SNPs contributing independently to PCa risk have been identified across populations through genome-wide association studies [5,6,7,8,9]. More recently, a large-scale meta-analysis of genotypes from more than 140,000 men has identified 63 novel PCa susceptibility loci [10].

Due to widespread screening for prostate specific antigen (PSA), early detection of PCa became possible. The majority of newly diagnosed cases involve localized (within the prostate) or regionally confined (spread into immediate surrounding tissue) tumors. In early stages of the disease, surgery and radiation can be curative with >99% 5-year relative survival [11]. However, about one-third of patients develop biochemically recurrent disease (rising PSA levels), which is treated with androgen deprivation therapy (ADT) when radiotherapy after prostatectomy is unsuccessful [12]. Since PCa cells are highly dependent on androgen signaling, ADT can delay disease progression. However, after initial therapy response, patients eventually become refractory to ADT. At this point, the disease has progressed to castration-resistant PCa (CRPC), which oftentimes develops concurrently with metastasis. CRPC is treated with androgen blockade and/or chemotherapy, which allows approximately 50% of patients to survive [13]. Still, despite remarkable progress in approved PCa treatment modalities in recent years, systemic therapies can only prolong survival for a few months [14].

Personalized medicine has the potential to maximize the response to therapies in cancer patients. In contrast to other frequent cancer entities, particularly melanoma, breast, lung, and colorectal cancers [14], such approaches are presently not fully established in PCa. This is primarily due to the paucity of specific biomarkers and therapeutic targets in the past. However, in recent years, large-scale genomic sequencing projects have catalogued common genomic alterations, which enabled an improved molecular classification of prostate tumors and led to a better understanding of the molecular mechanisms driving the initiation and progression of the disease. The clinical translation of these findings could pave the way towards the implementation of a personalized PCa therapy.

## 2. The Genomic Landscape of Primary Prostate Cancer

**Figure 1:**
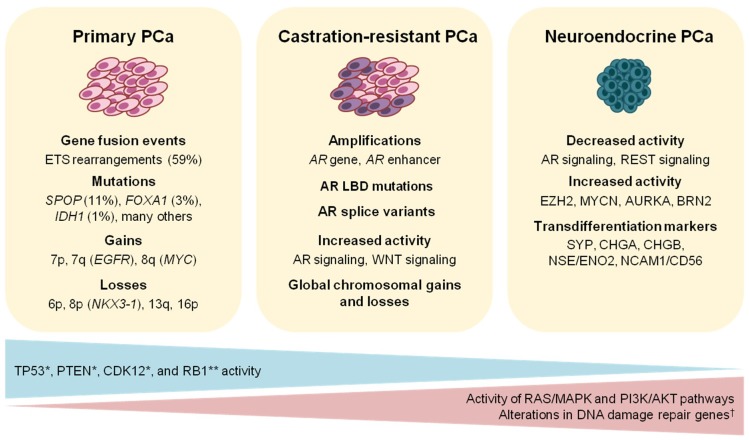
Characteristic molecular alterations in different stages of prostate cancer. Inactivation is markedly observed in castration-resistant (CR) * and neuroendocrine (NE) ** tumors. ^†^ Genetic alterations in the DNA damage repair pathways are increased in CR but are almost absent in NE tumors. AR = androgen receptor; LBD = ligand binding domain.

Genome sequencing studies in PCa have revealed many recurrent DNA alterations leading to deregulated biological processes involved in prostate development, cell-cycle regulation, androgen signaling, and chromatin organization, among others [15,16,17]. From these data, it has also been established that PCa is a cancer entity with a relatively low mutational burden of approximately 1 mutation per megabase [18]. On the other hand, multiple recurrent chromosomal gains and losses are common in PCa genomes [15,19]. Losses of chromosomes 6p, 8p, 13q, and 16p are considered early events in prostate oncogenesis [17]. These events result in the inactivation of tumor suppressor genes such as *NKX3.1* and *RB1* (Figure 1). Focal alterations of chromosomal loci spanning *PTEN*, *TP53*, and *CDKN1B*, among others, also contribute to the somatic copy number alteration (SCNA) pattern characteristic of PCa [15,17,18,19]. In parallel, the most frequent genomic gains in PCa occur on chromosomes 7 and 8q, the latter of which spans the *c-MYC* oncogene. Genome-wide SCNA has been reported to be an effective prognostic marker of PCa recurrence and metastasis. The CNA burden, quantified as the percent of autosomal tumor genomes harboring CNAs, positively correlated with disease progression, with a mean value of 4–5% in primary vs. 32% in metastatic tumors [19]. The ability of global SCNA levels to predict the risk of disease progression was exemplified by measuring the CNA burden and monitoring disease relapse over time in two cohorts of PCa patients at intermediate-risk (i.e., Gleason score 7). These tumors had a wide range of CNA burden (0.05–25% and 0.003–50%, respectively), suggesting the potential utility of this parameter to fine-tune the current risk stratification criteria. In both cohorts, CNA burden was significantly associated with biochemical recurrence and metastasis [19]. Whole genome doubling (WGD) also occurs in primary PCa, and this event is associated with the acquisition of CNAs during disease progression. In a prospectively sequenced cohort of primary tumors, WGD correlated with the Gleason grade. In a pan-cancer analysis, WGD directly corresponded with adverse patient survival. These observations imply the prognostic value of WGD as it emerges at a relatively early point in the course of cellular transformation [20].

Apart from large chromosomal gains and losses, several distinct genomic alterations are also present in PCa. Based on an integrative clustering approach using somatic mutations, gene fusions, SCNA, gene expression, and DNA methylation in 333 primary PCa samples, The Cancer Genome Atlas (TCGA) project defined seven major subtypes [18]: Tumors harboring fusions involving E-twenty-six (ETS) genes comprised the majority of the cohort (59%), with (i) 46% *ERG* rearrangements, followed by (ii) *ETV1* (8%), (iii) *ETV4* (4%), and (iv) *FLI1* (1%). Fifteen percent of the primary tumors carried mutations in either (v) *SPOP* (11%), (vi) *FOXA1* (3%), and (vii) *IDH1* (1%). In general, ETS fusion status highly affected primary PCa transcriptome as revealed by mRNA expression profiling. *FOXA1*- or *SPOP*-mutant tumors exhibited the highest AR transcriptional activity among all subtypes and harbored tightly correlated DNA methylation patterns of the most variable CpG sites. One of four generated methylation clusters was almost exclusively associated with the presence of the ERG fusion, while a CpG island methylator phenotype was attributable to *IDH1*-mutant tumors. Twenty-six percent of tumors remained unclassified, suggesting that their development had been driven by unknown molecular events, or a unique combination of genetic alterations. Indeed, in a larger sequencing cohort of 1013 local non-indolent PCa tumors, a total of 97 additional putative driver genes were reported to be mutated at frequencies below 3% [21]. Although the roles of these genes in PCa are still awaiting validation and functional analysis, their presence argues for the existence of a certain landscape of PCa characterized by low-frequency driver mutations. Because of the prevalence of the disease, even these low frequency mutations can potentially be useful for patient stratification.

### 2.1. ETS Fusion-Positive Prostate Cancer

Genomic rearrangements resulting in the androgen-driven expression of ETS family members represent the most recurrent molecular alteration in the PCa genome. Such gene fusions are clonal and considered to be early events in PCa development [22,23]. *TMPRSS2* and *SLC45A3*, which are normally regulated by the AR in the healthy prostate, are the common 5′ fusion partners of ETS genes. The current model for the initiation of the fusion event involves the introduction of double-strand breaks (DSBs) by liganded AR to specific AR binding sites. In the presence of genotoxic stress such as inflammation or infection, the activated AR recruits DNA-associated enzymes generating DSBs, which are repaired through non-homologous end joining, yielding the translocation products [24]. 

In PCa, the *TMPRSS2:ERG* (T2E) rearrangement on chromosome 21q is the most frequent variant among the possible gene fusion combinations. Despite the recurrent nature of this genomic alteration, ETS gene fusions alone are not sufficient to initiate tumorigenesis; other events such as *PTEN* loss or phosphatidylinositol 3-kinase (*PI3K*)*/AKT* activation must concomitantly be present for cellular transformation to occur [25,26]. ERG is a master transcription factor that interacts with several other cofactors to regulate the expression of target genes and modulate multiple biological processes that favor oncogenic transformation [27,28,29,30]. ERG induces the expression of the metalloproteinase *MMP3* and the plasminogen activator genes *PLAT* and *PLAU*, facilitating invasion of the T2E-positive PCa cell line VCaP [30]. In LNCaP T2E cell models, overexpression of the fusion resulted in the activation of the TGF-β signaling pathway and induction of epithelial to mesenchymal transition [29]. Moreover, by binding to the AR promoter region, ERG hinders AR-mediated, cell-specific differentiation of prostate cells [28]. ERG activation also perturbs the chromatin landscape of cells both in vitro and in primary tumors, which likely stems from the implementation of a T2E-specific transcriptional profile through co-option of other master transcription factors like HOXB13 and FOXA1 [31,32]. Notwithstanding the compelling evidence for the functional effects of the T2E fusion in PCa cells, its role in PCa formation and progression in vivo remains unclear. A positive association between T2E and aggressive PCa—as measured by elevated serum PSA or PCa specific death—has been observed in several studies, suggesting prognostic utility [33,34,35]. However, other groups did not find such association [36,37].

Multiple studies indicate the diagnostic relevance of using a combined assessment of T2E with either urinary non-coding RNA *prostate cancer antigen 3* (*PCA3*) or serum PSA levels [38,39] (see below). Urinary T2E transcripts from a cohort with high serum PSA and recommendation for biopsy and/or prostatectomy correlated with tumor size, high Gleason score at prostatectomy, and upgrading of Gleason grade following prostatectomy [35]. In a cohort of 291 men with elevated serum PSA, who were candidates for active surveillance, a correlation was established between diagnosis and high concentrations of urinary T2E and *PCA3* [40]. In a multicenter prospective study, assessment of urinary T2E in addition to PCA3 as predictive markers for PCa resulted in an increased detection sensitivity from 68% to 76% [41]. The same study also asserts that this dual marker approach could potentially reduce the number of men selected for prostate biopsy. In a radical prostatectomy cohort, significantly shorter times to recurrence were observed in patients with high T2E expression and elevated preoperative PSA [42]. Thus, there appears to be an opportunity for a better risk stratification strategy upon the addition of T2E and PCA3 to the standard of care approach. Indeed, diagnostic assays for T2E/PCA3 to predict high grade PCa in urine samples are in clinical use (Table 1).

### 2.2. ETS Fusion-Negative Prostate Cancer

Missense mutations in *SPOP* (Speckle-type POZ protein) are the most common point mutations in PCa, occurring in 10% of primary and metastatic tumors [17,18]. Loss-of-function *SPOP* mutations are mutually exclusive of ETS rearrangements, and *SPOP*-mutant tumors present distinct SCNA signatures, including *CHD1* deletion and losses on chromosomes 6q and 2q [18]. The *SPOP* gene codes for the substrate recognition component of a CUL3-based E3 ubiquitin ligase, which modulates the DSB repair machinery in a manner similar to BRCA1 [43]. Expression of oncogenic *SPOP*-mutant F133V in a conditional mouse model with Pten null background resulted in the formation of prostate tumors, in part through activation of the PI3K/mTOR signaling and upregulation of a network of AR-associated transcription factors [44]. In zebrafish, *SPOP*-mutants were shown to favor the error-prone non-homologous end joining (NHEJ) pathway upon DSB induction leading to increased intra-chromosomal rearrangements and genomic instability [43]. Since cancer cells with defects in DSB repair pathways are particularly sensitive to poly (ADP-ribose) polymerase (PARP) inhibitors, the same study tested the potential synthetic lethality of *SPOP* inactivation with these drugs in vitro: siRNA-based downregulation of *SPOP* or ectopic expression of its inactive mutant forms indeed lead to decreased cell viability upon PARP inhibition with olaparib [43]. These findings suggest that *SPOP* mutations could potentially become indicators for a subset of PCa amenable to PARP inhibition therapy.

Recurrent mutations in the forkhead transcription factor *FOXA1* gene are also found in primary PCa. FOXA1 targets include the *AR* gene, and consequently the transcription factor has been implicated in both AR-dependent and -independent roles in PCa oncogenesis [45,46]. *FOXA1* alterations in PCa are predominantly missense mutations located in the winged-helix domain of the protein. Those *FOXA1*-mutants preserve their DNA-binding capacity, which suggests that the functional impact of the mutations most likely stems from perturbed interactions with other chromatin-associating factors [18]. Interestingly, AR signaling activity was elevated in *FOXA1-*mutant primary tumors. This presents the possibility of using *FOXA1* mutational status as an indicator of a favorable clinical outcome upon ADT, at least in the context of castration-sensitive primary tumors [46].

*IDH1*-mutant tumors comprise a rare molecular subclass of primary PCa. The *IDH1* gene encodes the cytoplasmic form of isocitrate dehydrogenase that, upon mutation, becomes impaired and results in a novel enzymatic function of α-ketoglutarate conversion to 2-hydroxyglutarate (2-HG) [47]. 2-HG is involved in multiple cellular processes including hypoxia, establishment of histone modifications, and DNA methylation. The resulting epigenetic dysregulation leads to aberrant gene expression in cancer. Moreover, it has been recently reported that 2-HG shed by cancer cells suppresses T-cell activity, which promotes immune surveillance evasion in the tumor microenvironment [48]. In the TCGA cohort, *IDH1* R132 mutant tumors presented characteristics of early-onset disease, particularly lower SCNA burden and fewer genetic changes [18]. In gliomas and several hematologic malignancies, somatic *IDH1* and *IDH2* mutations have been associated with a characteristic DNA hypermethylation phenotype [49]. *IDH1-*mutant primary PCa tumors also manifested a similar phenotype, with levels of hypermethylation exceeding those of glioma or acute myeloid leukemia *IDH1*-mutant tumors [18]. PCa tumors with *IDH1* mutations are potentially clinically actionable due to their apparent clonal properties, as well as the druggability of the enzyme with synthetic inhibitors and immunotherapeutics [50,51].

### 2.3. Cellular Processes Deregulated in Prostate Cancer

Beyond the class-defining genetic changes discussed thus far, primary PCa can harbor multiple mutations in genes involved in PI3K/AKT and RAS/MAPK signaling, and DNA repair pathways [18,21,52] (Figure 1). The PI3K/AKT pathway is crucial for the regulation of cell proliferation, survival, and invasion. Aberrant activation of this pathway is found in 25–70% of PCa cases [52]. In addition to chromosomal loss and truncating mutations in the tumor suppressor *PTEN*, multiple activating genes of the PI3K pathway, including *PIK3CA, PIK3CB,* and *AKT1,* are mutated at low-frequencies [18]. More recently, oncogenic mutations in *PIK3R1* and *PIK3R2*, which encode PI3K regulatory subunits, were also reported in a large PCa exome-sequencing cohort [21]. 

The mitogen-activated protein kinase (MAPK) pathway has a central function in regulating cell proliferation. Although its hyperactivation in PCa is less established when compared to other tumor entities, activating mutations in genes of the RAS/MAPK pathway (e.g., *KRAS*, *HRAS*, and *BRAF*) have been identified in prostate tumors [18,21]. *BRAF*-mutated PCa is of clinical interest due to its potential sensitivity to MEK inhibitors [53,54].

Inactivation of DNA damage repair (DDR) pathway genes via somatic mutations is a frequent event in localized and, even more, in metastatic PCa [55]. DNA repair can be classified as targeting either single-strand breaks (SSBs) or DSBs. SSBs are corrected by mismatch repair (MMR), nucleotide excision repair (NER), and base excision repair (BER) mechanisms, whereas DSBs are repaired by NHEJ and homologous recombination (HR). In primary PCa, the collective genetic alterations of genes involved in these pathways were reported to occur in 10–20% of cases [18,21]. Most of these were found in *BRCA1*, *BRCA2*, *ATM*, *CDK12*, *FANCD2*, and *RAD51C*, all of which play crucial roles in the HR-mediated repair pathway. The HR repair is a high-fidelity process that requires a sister chromatid template, and thus is only active during the S and G2 phases of the cell cycle. Inactivation of *MLH1* and *MSH2*, which are key MMR genes, is also observed at low frequencies in primary PCa [55].

## 3. Current Molecular Biomarkers for Prostate Cancer Diagnosis and Risk Stratification

PSA screening was approved by the U.S. Food and Drug Administration (FDA) in 1994. Since then, the PSA test, together with digital rectal examination (DRE), has become the standard means for identifying men who should be recommended to undergo prostate biopsy. From the early 1990s until recently, PSA testing was implemented as a population-wide screening test in the U.S. [56]. The use of PSA testing continues to be controversial. While on one hand this diagnostic approach allows for accurate recruitment of patients for biopsy in the majority of cases, a considerable number of uncertain findings remain, leading to unnecessary initial and repeat biopsies, overdiagnosis, and overtreatment with inevitable side effects [5]. Consequently, the U.S. Preventive Services Task Force (USPSTF) indicated an age-dependent recommendation for PSA testing in men [57]. In addition, full disclosure of the advantages and drawbacks of the test must be given to the concerned individuals. This guideline aims to encourage individualized administration of the test and to promote a shared decision-making approach that might mitigate the aforementioned risks of population-wide screening [58]. The guidelines of the European Association of Urology-European Society for Radiotherapy and Oncology-International Society of Geriatric Oncology (EAU-ESTRO-SIOG) recommend PSA testing in men with elevated risk of PCa [59]. 

Novel diagnostic biomarkers in blood and urine have been developed in recent years with the aim of augmenting PSA test results and guiding physicians in deciding which patients should undergo initial biopsy or re-biopsy (Table 1). The non-coding RNA *PCA3* is overexpressed in 95% of primary and metastatic PCa tissue samples [60,61]. The FDA-approved PCA3 assay is performed by measuring *PCA3* levels in the urine after prostatic massage and normalizing the value with PSA. The urine assay ExoDx^®^ prostate intelliscore (Exosome Diagnostics, Boston, MA, USA) is a reverse transcription quantitative polymerase chain reaction (RT-qPCR)-based test that measures urine exosome-derived *PCA3* and T2E transcript levels [38]. The Mi-Prostate Score urine test (University of Michigan, Ann Arbor, MI, USA) applies the T2E gene fusion status in combination with PSA and PCA3 levels to a validated logistic regression model, generating the Mi-Prostate Score (MiPS), which provides improved PCa prediction with an AUC of 0.88, 90% specificity, and 80% sensitivity [62]. SelectMDx (MDxHealth, Irvine, CA, USA) is a post DRE-urine test that assays the mRNA levels of *DLX1* and *HOXC6*. A low-risk SelectMDx score correlates with 90% probability of a negative diagnosis [63]. The 4Kscore^®^ Test (OPKO Diagnostics, Woburn, MA, USA) or the four-kallikrein panel is a commercially available prebiopsy blood test that is used in combination with clinical information (i.e., age, race, DRE, PSA, and family history) for predicting the risk of aggressive PCa after a biopsy is performed [64]. The test measures three different PSA isoforms: total (tPSA), free (fPSA), and intact (iPSA), and human kallikrein-related peptidase 2 (hK2). The Prostate Health Index (PHI, Beckman Coulter, Brea, CA, USA) is a FDA-approved quantitative kallikrein immunoassay that quantifies tPSA, fPSA, and [−2]proPSA (p2PSA) into a single numerical score (PHI score: p2PSA/fPSA × √tPSA). It has been shown to improve PCa detection 3-fold compared with PSA testing alone [65]. The ConfirmMDx Prostate Cancer test (MDxHealth, Irvine, CA, USA) was developed to address false-negative biopsy histopathology concerns. This PCR-based panel quantifies methylated CpG islands of the tumor suppressor genes *GSTP1*, *APC*, and *RASSF1*. A meta-analysis revealed that *GSTP1* is hypermethylated in up to 90% of PCa cases, and its specificity for PCa has been validated in multiple studies [66]. *APC* and *RASSF1* serve as field effect markers that detect transformed cells beyond morphologically distinct foci [67]. 

Risk assessment of PCa is of paramount importance for clinical management and treatment decisions. The standard risk stratification scheme incorporates clinical information including minimum of stage, grade, and PSA level to classify cases into five risk categories [68,69]. Table 1 lists clinically validated molecular platforms used for PCa prognostication and risk stratification. NaDiA^®^ ProsVue^TM^ (Beckman Coulter, Brea, CA, USA) is an ultrasensitive immuno-PCR assay that quantifies total serum PSA [70]. In a prospective-case cohort, the ProsVue PSA slope readout has been determined to be prognostic of clinical recurrence and PCa-specific mortality [71,72]. In addition, four tissue-based molecular tests have been included in the 2018 National Comprehensive Cancer Network Guidelines for PCa [73,74]. Decipher^TM^ (GenomeDx Biosciences, Vancouver, BC, Canada) is a transcriptomic microarray that profiles the expression of 22 gene markers from formalin-fixed paraffin embedded (FFPE) prostate tissue. A Genomic Classifier (GC) score is generated based on the resulting expression signature. Higher GC scores correlate with tumor aggressiveness and patients with elevated scores were reported to experience earlier death from PCa [75]. Oncotype DX^®^ (Genomic Health, Redwood City, CA, USA) is a quantitative RT-qPCR based assay that measures the expression level of a panel of 12 PCa-related genes and 5 housekeeping controls from FFPE tissue samples. The numeric output of the test, termed Genomic Prostate Score (GPS), is associated with clinically aggressive disease [76,77]. Prolaris^®^ (Myriad Genetics, Salt Lake City, UT, USA) is another quantitative RT-qPCR-based assay measuring the expression of a panel of 31 cell cycle-related genes and 15 housekeeping controls from FFPE tissue samples. From the resulting expression analysis, a cell cycle progression (CCP) score is generated wherein each unit increment corresponds to an expression level doubling, indicating poorer prognosis of the disease [78,79]. The ProMark^®^ (Metamark, Cambride, MA, USA) test is a quantitative multiplex proteomics in situ imaging-based panel that assesses an 8-protein signature from FFPE biopsy tissue and generates a risk score [80]. Validation of the assay revealed significant correlation between the generated risk score with aggressive disease and lethal outcome [80,81].

Although the liquid and tissue-based tests provide useful diagnostic and prognostic information, they have limitations: Mutational processes during tumor development result in extensive heterogeneity of PCa both within a tumor (intra-tumor) and between different tumors (inter-tumor). Thus, there is a considerable risk of undersampling, which devalues the significance of most tissue-based biomarkers. Benign tissue close to cancerous lesions could also manifest molecular changes, and such field effects might affect the testing outcome [93]. 

## 4. Prognostic Molecular Marker Signature in Prostate Cancer

Considering the low number of genetic changes with prognostic significance in primary PCa, a pioneering study used the entire PCa genome to generate a signature of molecular events correlated with disease relapse and patient survival [15]. This large-scale analysis utilized a cohort of localized, non-indolent PCa comprised of 200 whole genome and 277 whole exome sequences. A low frequency of single-nucleotide variants (SNVs) was found, with a median of 0.53 somatic SNVs per megabase. Only six genes (*SPOP*, *TTN*, *TP53*, *MUC16*, *MED12*, and *FOXA1*) carried coding mutation frequencies greater than 2%. In this study, T2E fusions were detected in 38% of the tumors, and localized somatic hypermutation events, particularly kataegis and chromothripsis, were observed in 20% and 23% of the samples, respectively. While kataegis was associated with Gleason score, and chromothriptic events increased in frequency with tumor size, both hypermutation events did not correlate with other clinical parameters. The DNA repair gene *ATM* was mutated in 1.75%, and the corresponding SNV was indicative of disease relapse. Moreover, in this cohort, an interchromosomal translocation event centromeric on chromosome 7 and an amplification of the *MYC* gene were predictive of biochemical recurrence. On the epigenetic level, hypermethylation of the transcriptional elongation regulator *TCERG1L* was associated with a poor clinical outcome. Finally, the multi-modal biomarker panel for disease relapse prediction generated from this meta-analysis (AUC = 0.83, concordance index = 0.79) was comprised of the tumor stage, *ACTL6B* and *TCERG1L* methylation, the chromosome 7 breakpoint, SNVs in *ATM*, and *MYC* CNA [15]. 

An interesting insight gained from the same study is the prognostic impact of aberrant focal DNA methylation. The methylation status of selected genes was more tightly correlated with disease recurrence than other genomic characteristics: six out of the nine events that significantly associated with disease outcome pertained to DNA methylation status [15]. This observation is in line with several reports emphasizing the suitability of these epigenetic modifications as markers for early disease detection and outcome prediction [66,87,94]. 

## 5. Treatment of Localized Prostate Cancer

**Figure 2:**
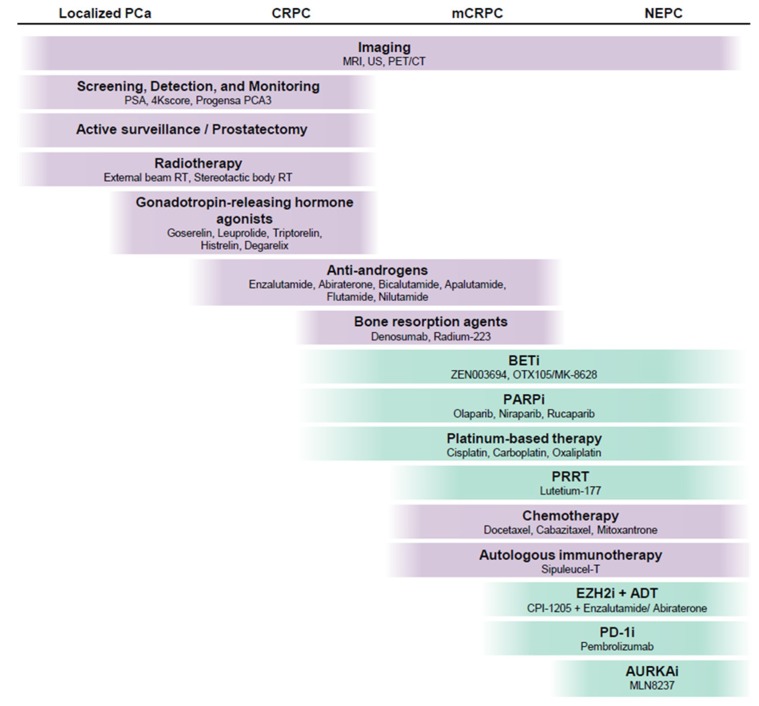
Management options with selected therapeutic agents for localized and advanced prostate cancer. Purple bars denote treatments with regulatory approval; green bars denote therapeutic agents in clinical trials. PCa = prostate cancer; CRPC = castrate-resistant PCa; mCRPC = metastatic CRPC; NEPC = neuroendocrine PCa; MRI = magnetic resonance imaging; US = ultrasound; PET/CT = positron emission tomography/computed tomography; BETi = BET inhibition; PARPi = PARP inhibition; EZH2i = EZH2 inhibition; PD-1i = PD-1 inhibition; and PRRT = peptide receptor radionuclide therapy.

To date, molecular markers have not yet altered the treatment concept in localized PCa. Expectant management, radical prostatectomy (RP), and radiotherapy are still the primary therapeutic options for men with localized PCa. Expectant management entails monitoring of the disease progression without undergoing therapy and can be further classified into watchful waiting and active surveillance. Watchful waiting involves treatment with palliative intent and is applicable to elderly or frail men with a high comorbidity [95]. For younger men diagnosed with low-grade (Gleason score of 6 or less) PCa, active surveillance is typically undertaken with the aim of delaying treatment onset and avoiding its side-effects until cancer progression. The patients are routinely monitored using serum PSA tests, prostate biopsies, and MRI [22]. RP is a standard treatment option for patients with localized disease (Figure 2). Due to potential risks associated with surgical procedures, the decision for surgery is taken in patients with a life expectancy of at least 10 years [96,97]. External beam radiation therapy (EBRT), stereotactic body radiotherapy (SBRT), brachytherapy, and proton therapy are among radiation techniques performed to treat localized PCa [74]. Other local therapeutic strategies that may be clinically indicated are cryotherapy and high-intensity focal ultrasound (HIFU). Cryotherapy is a minimally invasive procedure, which aims to damage the tumor tissue by local freezing. HIFU uses ultrasonic wave transmission to induce tissue destruction through physical and thermal means [98]. Both local therapies are recommended by the National Comprehensive Cancer Network [74] upon disease recurrence post radiotherapy while the European Association of Urology–European Society for Radiotherapy and Oncology–International Society of Geriatric Oncology Guidelines still consider these procedures as fully experimental and should only be offered within clinical trials [59].

AR signaling is fundamental for prostate cell growth and survival, and inhibiting AR activation is part of the therapeutic repertoire against PCa. ADT is a common treatment for biochemically recurrent disease post local curative salvage options. ADT can be implemented chemically with luteinizing hormone-releasing hormone (LHRH, or gonadotropin-releasing hormone, GnRH) agonists or antagonists, or surgically through bilateral orchiectomy (Figure 2). Both treatment regimens can be augmented with AR blockade [12]. The rationale of ADT implementation post-RP and pelvic lymphadenectomy, but prior to detectable metastatic lesions, is based on the Eastern Cooperative Oncology Group (ECOG) study in which patients who underwent ADT, either by administration of the LHRH agonist goserelin, or by bilateral orchiectomy had improved survival outcomes compared with patients in the observation group (hazard ratio = 1.84) [99]. Currently, the utility of the AR antagonist enzalutamide, an inhibitor binding covalently to the ligand binding domain (LBD)—alone or in combination with leuprolide, a LHRH agonist—in a similar setting (i.e., high-risk localized PCa post RP and/or radiation therapy) is being assessed in the ongoing EMBARK study (NCT02319837) [100].

## 6. Molecular Alterations and Current Therapy of Advanced Prostate Cancer

Seeding of metastases from the prostate to other parts of the body—most commonly to lymph nodes, bones, or lung—characterize advanced PCa [101]. Once PCa progresses to the metastatic state, therapy shifts from a localized approach into systemic chemotherapy and/or ADT (Figure 2). Administration of systemic therapies is currently still non-discriminative, that is, without prior patient stratification, and the optimal sequence and combination of treatments is still empirically determined, since large, randomized clinical trials addressing these parameters are lacking to date [102].

Docetaxel (first-line) and cabazitaxel (second-line) are taxanes that induce cell cycle arrest and disrupt nuclear translocation of AR by binding to and stabilizing microtubules [103]. Radium-223 (^223^Ra) is an α-particle emitter that selectively binds to osteoblastic PCa bone metastases. In comparison with a placebo group, ^223^Ra therapy increased median survival by 3.6 months and retarded the time to first skeletal-related lesion by 5.8 months [104]. A phase 3 clinical trial showed that Sipuleucel-T, an autologous cellular immunotherapy, provided overall survival benefit to patients with metastatic CRPC (mCRPC) [105]. Skeletal-related events are essential considerations upon treatment of mCRPC patients. Zoledronic acid, a highly potent inhibitor of osteoclast-mediated bone resorption, is the most established preventive therapy for men with recurrent and metastatic PCa [106]. Multiple clinical studies have shown that in comparison to zoledronic acid, denosumab, a fully humanized antibody targeting the receptor activator of nuclear factor κ-B ligand (RANKL), is a superior osteoprotective agent in mCRPC patients [107,108,109]. However, it remains unclear whether these drugs lead to improved overall patient survival [110].

In mCRPC, the most prominent genes affected by CNA are the *AR* (~63%) and the tumor suppressors *TP53* (~53%) and *PTEN* (~40%) [16]. Mutations in *ATM* (~7%), *BRCA1/2* (~14%), *AR,* and *TP53* are more frequent compared to primary PCa [18]. About 18% of advanced PCa cases harbor alterations associated with the WNT pathway, including hotspot mutations in *CTNNB1* or recurrent alterations in *APC*, leading to increased WNT signaling [16]. Focal amplification of *AR* and its enhancer elements contributes to castration resistance and is recurrent in advanced PCa (Figure 1) [111,112,113]. Recent whole genome analyses of mCRPC also revealed tandem duplications proximal to the *c-MYC* locus, resulting in the deregulation of long non-coding RNAs responsible for post-translational control of the c-MYC protein [114]. Rs11672691 at 19q13, associated with aggressive PCa, was identified as part of an enhancer element synergizing with HOXA2 to enhance expression of CEACAM21 and PCAT19 and promote tumorigenesis [115]. 

On average, advanced PCa harbors eight biologically relevant genetic aberrations in protein coding regions in addition to frequent copy number gains of 8q and losses of 8p, 13q, 16q, and 18q [16,116,117]. SNVs in potential driver gene fusions, driver homozygous deletions, and driver amplifications occur in 99% of mCRPC [16,118]. Next generation genomic sequencing and mapping detected 34 large structural variants (SVs), each longer than 1 kilobase in length, with suggestive functional oncogenic relevance [119]. *PTEN* deficiency occurs in approximately 50% of CRPC and is associated with decreased time to metastasis and poor prognosis, making *PTEN* a prognostic factor in combination with other markers [120]. Patients with a *PTEN* mutation or deletion might benefit from PARP inhibitor therapies (see above)—due to deficiencies in homologous recombination [121,122]—or combinational therapies with the AKT inhibitor ipatasertib and the anti-androgen abiraterone [123].

### Androgen Deprivation Therapy and Combined Androgen Blockade in Metastatic Prostate Cancer

ADT is considered to be a primary systemic therapy for men with metastatic PCa, as well as an adjuvant therapy concurrent with surgery or radiation therapy [74]. In 2015, the phase III CHAARTED and STAMPEDE clinical trials reported the effectiveness of the combined administration of ADT and docetaxel in hormone-sensitive metastatic disease [124,125]. Following this, in 2017 the combination of ADT and the high-affinity irreversible cytochrome P450 17A1 (CYP17A1) inhibitor abiraterone with a glucocorticoid has been established as a new standard of care for patients with metastatic PCa based on the results of the STAMPEDE and LATITUDE trials [126,127]. Abiraterone, enzalutamide, and apalutamide are hormonal agents that have had the largest impact in CRPC management. For both pre- and post-chemotherapy settings, administration of either abiraterone or enzalutamide resulted in improved overall survival in mCRPC patients [128,129,130,131]. The next-generation androgen inhibitor apalutamide, which received regulatory approval in February 2018, significantly prolonged metastasis-free survival and time to tumor progression in non-metastatic CRPC [132].

In the majority of cases, metastatic PCa eventually becomes unresponsive to ADT and progresses to castration resistance. CRPC can be mediated by (i) *AR* gene and/or *AR* enhancer amplification, leading to increased AR protein expression; (ii) AR-LBD mutations; or (iii) expression of *AR* splicing variant 7 (AR-V7) (Figure 1). Aside from gene amplification, AR protein overexpression in CRPC can be facilitated by tandem duplications of an enhancer element 650 kb upstream of the *AR* gene [112,113]. Gene and enhancer amplifications maintain increased AR signaling even under reduced agonist availability. Mutations in the AR-LBD decrease AR responsiveness to anti-androgens, and the AR can be activated by non-canonical ligands such as estrogen or antagonist-agonist switching [133]. AR-V7 is strongly associated with CRPC [134] and can be monitored by isolating circulating tumor cells (CTCs) from liquid biopsies [135]. AR-V7 is a C-terminus-truncated splicing variant that mediates ADT resistance due to its lack of the AR-LBD. The response of CRPC patients to apalutamide, enzalutamide [136], or abiraterone [128] indicates that castration-resistant tumors are still highly dependent on AR signaling and patients can benefit from continued ADT with increased dosage (Figure 2). 

## 7. Novel Approaches to the Classification and Treatment of Advanced Prostate Cancer

### 7.1. DNA Repair

There is considerable interest in exploiting the potential vulnerabilities of cancer cells harboring loss-of-function aberrations in DNA repair genes (e.g., *BRCA2*, *BRCA1*, and *ATM*), which are mutated in approximately 20% of mCRPC [16]. In ovarian and breast malignancies, PARP inhibition therapy against tumors within this molecular subclass is already established, and this provides a strong rationale to challenge prostate lesions with a similar genomic profile. PARP1/2 enzymes detect SSBs and aid in coordinating the SSB repair response. Upon PARP1/2 inhibition, unrepaired SSBs eventually lead to DSBs that become lethal to cells lacking an intact HR repair response [55,137]. To date, the most important application of olaparib, a pharmacological PARP inhibitor, in PCa has been in phase II clinical trials involving patients with advanced castration resistant disease [138,139,140,141]. In TOPARP-A, the initial stage of the Trial of PARP Inhibition in Prostate Cancer (TOPARP, NCT01682772 [141]) study, 50 patients received olaparib and were assessed for response to treatment according to Response Evaluation Criteria in Solid Tumors (RECIST), serum PSA decline, and/or reduction of CTC count. Among the 49 patients that could be evaluated, 16 (33%) responded to the treatment. A significant positive correlation between response and presence of mutations or homozygous deletions in DNA repair genes was identified using targeted exome sequencing [138]. The encouraging results of the TOPARP-A study led to a second trial (TOPARP-B), wherein patients were prospectively selected based on the presence of biomarkers predictive of olaparib response, particularly the genotypic status of *BRCA2* and *ATM*. This ongoing study aims to validate the potential of olaparib treatment in patients with inactivating mutations in these commonly affected DNA repair genes, as well as to assess the sensitivity of patients with mutations or losses in other less frequently affected genes [55]. The role of the T2E gene fusion in advanced PCa is still controversial. Tumors harboring the gene fusion show a two- to five-fold increased frequency of mutations in the *PTEN* coding region [142]. PARP inhibitors enhance ETS-induced DNA damage, inhibit ETS transcriptional activity, and reduce ETS-activated invasiveness of PCa cell lines [143]. Since PARP1 is a required ERG cofactor, and PARP inhibition increases the susceptibility of PCa cells to low-dose radiation, ETS fusion-positive tumors might respond to treatment with PARP inhibitors in combination with radiotherapy. However, a recently concluded biomarker-stratified and randomized phase II multicenter clinical trial in mCRPC patients revealed that ETS-fusion status does not predict response to a combination therapy of abiraterone and veliparib, a PARP1/2 inhibitor [144]. Indeed, the clinical significance of this concept for targeted therapy requires further evidence [143,145].

Platinum-based interstrand crosslinking compounds are similarly being examined as therapeutic agents in advanced PCa. Alone or in combination with conventional chemotherapy, these agents have been tested in medium sized clinical trials and have shown moderate anti-tumor activity in molecularly heterogeneous patients [146,147,148,149]. Significant toxicities have been reported for some combination therapies, however [150,151,152], and for the oral compound satraplatin, further efforts in drug development were halted after a large phase III trial failed to improve overall survival in mCRPC [153]. There has been a revival of interest in platinum compounds in light of recent findings that DDR genes are frequently altered in advanced PCa [154]. Reflecting this are two ongoing clinical trials (NCT02311764, NCT02598895) on carboplatin administration in mCRPC patients with defects in DDR pathway genes [155,156].

### 7.2. Immune Checkpoint Inhibition

A hallmark of cancer cells is their potential to evade the immune response [157]. Five to twelve percent of mCRPC are associated with MMR deficiency, usually due to mutations in *MLH1*, *MSH2*, and *MSH6* [16,158]. The FDA-approved drug pembrolizumab, a programmed cell death protein 1 (PD-1)-targeting antibody, has shown clinical utility against malignancies with MMR defects, particularly in melanoma, non-small cell lung cancer, and colorectal cancer [159,160]. Early phase II clinical trials in molecularly heterogeneous mCRPC patients reported response rates of 10–20% to pembrolizumab therapy [161,162]. Furthermore, inhibitors of PD-1 ligand 1 (PD-L1) are in clinical trials for several advanced cancers [163]. PD-L1 belongs to the CD28/B7 superfamily and inhibits T-cell mediated immune response. Monoclonal antibodies against PD-L1 stimulate the immune system by increasing T-cell activation. When the tumor relapses under ADT, elevated levels of PD-L1 positive dendritic cells can be measured in the blood of mCRPC patients [164]. Increased PD-L1 levels were also observed by immunohistochemistry in mCRPC compared to primary PCa [165]. Further studies are needed to fully establish the relationship between PD-1/PD-L1 blockade response and genomic status, and to explore the clinical potential of immune checkpoint inhibitors in PCa (Figure 2).

Biallelic loss of *CDK12*, which codes for an expression regulator of DNA damage response genes, is enriched in mCRPC compared to primary PCa [166]. *CDK12* alterations are mutually exclusive with other PCa drivers such as ETS fusions or *SPOP* mutations. Focal tandem duplications (FTD) of regions spanning cell cycle genes, and maintenance of BRCA1 or BRCA2 expression, characterize *CDK12*-deficient mCRPC. A recent analysis of 360 mCRPC samples revealed the impact of FTDs on neoantigen production in *CDK12*-deficient tumors, and this suggests immune checkpoint inhibitors as prospective targeted therapy for this class of mCRPC [166].

### 7.3. Epigenetic Alterations

Bromo- and Extra-Terminal domain (BET) proteins are epigenetic readers of acetylated lysines. This protein family includes bromodomain 2 (BRD2), BRD3, and BRD4, all of which regulate gene expression of inflammatory factors and cell cycle genes [167]. A variety of diseases and cancers are linked to BET proteins [168]. BET inhibitors (BETis) are recently developed anti-cancer agents that function at the epigenetic level for treating different tumor entities. BETi application is also a way to indirectly inhibit the protooncogene c-MYC [169]. Amplification of the c-MYC locus or elevated expression levels promotes tumorigenesis of mCRPC [114,170,171,172]. Furthermore, BETi JQ1 inhibits the recruitment of AR to its target loci by interrupting the physical interaction of BRD4 with the N-terminal AR domain. Thus, BETis act downstream of AR to inhibit androgen signaling and PCa progression [173,174]. In preclinical settings, it has been shown that *SPOP*-mutant PCa cells are less responsive to BETi treatment. SPOP labels BRD4 for ubiquitination-mediated degradation and, consequently, *SPOP*-deficient tumors present elevated BRD4 levels. This overexpression explains how these tumors can tolerate BET inhibition [175]. One possible option to improve the outcome of BETi treatment in *SPOP*-mutant CRPC is a combinational therapy with AKT inhibitors to block the BRD4-mediated activation of AKT–mTORC1 signaling [176]. Currently, there are different BETis (ZEN003694 or OTX105/MK-8628) in clinical trials (NCT02705469 [177] and NCT02259114 [178]) for CRPC and mCRPC (Figure 2).

### 7.4. Molecular Markers of Neuroendocrine Prostate Cancer

Small cell or neuroendocrine PCa (NEPC) is a highly aggressive subtype that arises in less than 2% of untreated cases (i.e., *de novo* NEPC) [179]. A highly similar histologic subtype has also been observed in tumors that have become resistant to AR-targeting therapy (i.e., treatment-emergent NEPC) [180]. NEPC is distinguished from prostatic acinar carcinoma by unique clinical and ultrastructural characteristics, as well as positive immunohistochemical staining for various neuroendocrine markers and polypeptide hormones [181] including synaptophysin (SYP), chromogranins A and B (CHGA, CHGB), neuron-specific enolase (NSE/ENO2), or neuronal cell adhesion molecule (NCAM1/CD56) [181,182]. This PCa subtype is clinically defined by rapid disease progression, recurrent lytic bone lesions, frequent metastatic spread to visceral organs, and poor survival [183,184]. It was initially assumed that NEPC developed from outgrowth of normal prostatic neuroendrocrine (NE) cells, promoted by the selective pressure of androgen-independent growth [185]. Current evidence, which is supported by clonal molecular events such as T2E gene fusion and *TP53* mutations [186,187,188], suggests a common origin of adenocarcinoma and NEPC. Furthermore, the extent of NE differentiation correlates with exposure to ADT and represents an adaptive clinical phenotype, which is of special significance considering the advent of novel and highly potent AR-targeted therapies [180,189,190]. Androgen-deprivation induced a neuronal morphology and expression of NSE and CHGA in LNCaP cells [191]. Concurrent loss of *RB1* and *TP53* is significantly increased in NEPC (~53%) compared to CRPC (~14%) [180,192], leading to enhanced lineage plasticity, which promotes a shift to a basal-like AR-signaling independent subtype and augmented metastasis. RB1 dysfunction causes deregulation of epigenetic factors, including SOX2 and EZH2. Combination therapy of EZH2 inhibitors with the anti-androgen enzalutamide significantly reduced tumor growth in mouse models compared to enzalutamide treatment alone [117]. Therapy with enzalutamide/abiraterone combined with an EZH2 inhibitor (CPI-1205) is currently being tested for patients with mCRPC (Figure 2, NCT03480646 [193]).

Further molecular features of NEPC are attenuated AR signaling, decreased RE1-silencing transcription factor (REST) signaling [194], elevated EZH2 levels [195], and increased *N-MYC* and Aurora kinase A (*AURKA*) expression (Figure 1). A recent analysis of 249 metastatic biopsies from 202 patients revealed a 17% prevalence rate of tumors presenting small cell features congruous with NEPC. Unsupervised clustering of transcriptional profiles in combination with histopathological evaluation and AR immunohistochemistry presented a wide spectrum of molecular alterations in NEPC. Deleterious mutations in DDR pathway genes identified via somatic targeted sequencing were almost absent in NE-positive tumors (*p* = 0.035) [196], despite their enrichment in CRPC [16]. 

AR deactivation promotes the expression of BRN2 (encoded by *POU3F2*), a neuronal transcription factor and a major driver of NE differentiation [197]. AURKA interacts with the transcription factor N-MYC, whose overexpression in LNCaP cells results in increased binding to the promoters of *NSE* and *SYP* [179]. In turn, AURKA inhibition reduced the viability of N-MYC-overexpressing LNCaP cells and induced tumor shrinkage in NE-tumor xenograft models, with concomitant decrease in SYP expression [179]. In a mouse transplantation model of human prostate basal epithelial cells overexpressing N-MYC, activation of AKT1 downstream of N-MYC was sufficient to induce NEPC as detected by positive immunohistochemical staining of NE markers (i.e., CHGA, SYP, NCAM1, and NSE) and concurrent abolition of the AR [198]. Detection of *AURKA* amplification and overexpression in primary PCa specimens suggests its potential as a marker to identify patients who are more likely to develop NEPC [179]. N-MYC overexpression reduces AR expression, and this could favor the development of an AR independent state [179]. Sustained N-MYC expression combined with AKT1 signaling is necessary for tumor maintenance. One approach to target N-MYC is by inhibiting the stabilizing AURKA to allow for the ubiquitination of N-MYC by the E3-ligase FBXW7 for proteosomal degradation [199]. Since CRPC and NEPC show increased abundance of AURKA [200,201] (Figure 2), the AURKA inhibitor alisertib is currently being assessed as therapy for mCRPC and NEPC in an ongoing phase II study (NCT01848067 [202]). 

Finally, another treatment option for NE tumors is peptide receptor radionuclide therapy (PRRT) using radioisotopes. PRRT is a personalized therapy in which cytotoxic levels of radiation are shuttled by a peptide to the tumor cells [203]. PRRT is currently in a clinical trial (NCT03042312 [204]) for NEPC (Figure 2).

## 8. Summary

At the time of diagnosis, PCa frequently manifests as an organ-confined disease, and most tumors present indolent features [205,206]. Nonetheless, a sizeable proportion of cases progress with highly variable clinical courses that often precede a lethal outcome. Integrative analyses of large-scale sequencing data from the TCGA and the International Cancer Genome Consortium (ICGC) have enabled a molecular taxonomy of clinically heterogeneous prostate tumors that can potentially be useful for patient stratification. Primary PCa can be broadly classified as to harboring ETS gene fusions, or mutations in *SPOP*, *FOXA1*, and *IDH1*, as well as other drivers at low frequencies. PCa progression is accompanied by deregulation of a number of biological processes including PI3K/AKT1 signaling, RAS/MAPK signaling, and DNA damage repair pathways. Advanced castration-resistant PCa is molecularly defined by genetic alterations converging on AR signaling. These alterations also rationalize the capability of CRPC tumors to tolerate androgen deprivation. In contrast, NEPC is characterized by an almost complete abrogation of AR activity. The molecular etiology of the NE subtype is presently unclear, but these tumors are characterized by overexpression of transdifferentiation markers and increased N-MYC activity through stabilization by AURKA.

In the future, molecular markers may complement PCa theranostics, in a manner similar to other tumor entities such as melanoma, colorectal, breast, and lung cancers [207,208,209]. Simultaneously, the broad spectrum of novel therapeutic targets recently reported in advanced PCa presents the possibility for personalized medicine strategies for patients with metastatic disease (Figure 2) [210]. Several promising treatment concepts including PARP inhibition and epigenetic therapy are currently being explored in clinical trials, and other options are likely to emerge in the next years. Not unexpectedly, the same projects that generated genomic, transcriptomic, and epigenomic stratification markers from thousands of patients have also corroborated the notion that PCa is a molecularly complex disease. Recent findings add novel perspectives to the underlying molecular and cellular processes in PCa development and therapy. A case-in-point is the current report that non-coding genomic alterations (e.g., structural rearrangements in cis-regulatory elements) in PCa and in other tumor entities play much more prominent roles in aggressive cancers than previously anticipated [112,113]. Ultimately, functional characterization of these events will improve our current knowledge of PCa.

Despite the encouraging developments, current clinic-pathological criteria are still insufficient to accurately distinguish between indolent and aggressive PCa. The identification of biomarkers predicting clinically aggressive PCa and metastatic dissemination remains a challenge and should be a priority for future PCa genome research. 

## Figures and Tables

**Table 1:** Prostate cancer diagnostic and prognostic biomarkers.

Test	Company	Sample Input	Testing Platform	Outcome	References
*Clinically available biomarkers for diagnostic assessment*					
ExoDx^®^	Exosome Diagnostics	Urine	Transcript quantification of *PCA3* and *TMPRSS2:ERG*	Initial biopsy	[38,39]
Mi-Prostate Score	University of Michigan	Post-DRE urine	Transcript quantification of *PCA3* and *TMPRSS2:ERG*, in combination with PSA level	Initial biopsy	[82,83]
SelectMDx	MDxHealth	Post-DRE urine	Transcript quantification of *DLX1* and *HOXC6*	Initial biopsy	[63]
Progensa PCA3	Hologic	Post-DRE urine	Transcript quantification of *PCA3* and *PSA*	Rebiopsy	[60,61]
4Kscore^®^	OPKO Diagnostics	Blood	Immunoassay panel for tPSA, fPSA, iPSA, and hK2	Rebiopsy	[64,84,85,86]
ConfirmMDx	MDxHealth	Prostate tissue	PCR-based determination of the methylation status of *GSTP1*, *APC*, and *RASSF1*	Rebiopsy	[66,67,87,88]
Prostate Health Index	Beckman Coulter	Blood	Immunoassay panel for tPSA, fPSA, and p2PSA	Initial biopsy/rebiopsy	[65,89,90]
*Molecular panels for prognosis and risk stratification*					
NaDiA^®^ ProsVue^TM^	Beckman Coulter	Blood	Total serum PSA	Management post-RP	[70,71]
Decipher^TM^	GenomeDx Biosciences	RP	Transcriptomic microarray profiling of 22 gene markers	Management post-RP	[75,91]
Oncotype DX^®^	Genomic Health	Prostate tissue	Quantitative PCR for 12 PCa-related genes and 5 housekeeping controls	Active surveillance or treatment initiation	[76,77]
Prolaris^®^	Myriad Genetics	Prostate tissue	Quantitative PCR for 31 cell cycle-related genes and 15 housekeeping controls	Active surveillance or treatment initiation	[78,79]
ProMark^®^	Metamark	Prostate tissue	*In situ* measurement of 8 protein biomarkers by automated image analysis	Active surveillance or treatment initiation	[80,81,92]

PSA = prostate-specific antigen; tPSA = total PSA; fPSA = free PSA; p2PSA = [−2]proPSA; DRE = digital rectal examination; RP = radical prostatectomy.

## References

[1] Ferlay J., Soerjomataram I., Dikshit R., Eser S., Mathers C., Rebelo M., Parkin D.M., Forman D., Bray F. (2015). Cancer incidence and mortality worldwide: Sources, methods and major patterns in GLOBOCAN 2012. Int. J. Cancer.

[2] SEER Cancer Statistics Review, 1975–2014. https://seer.cancer.gov/csr/1975_2014/.

[3] Lee J., Demissie K., Lu S.E., Rhoads G.G. (2007). Cancer incidence among Korean—American immigrants in the United States and native Koreans in South Korea. Cancer Control.

[4] Zeigler-Johnson C.M., Rennert H., Mittal R.D., Jalloh M., Sachdeva R., Malkowicz S.B., Mandhani A., Mittal B., Gueye S.M., Rebbeck T.R. (2008). Evaluation of prostate cancer characteristics in four populations worldwide. Can. J. Urol..

[5] Thompson I.M., Pauler D.K., Goodman P.J., Tangen C.M., Lucia M.S., Parnes H.L., Minasian L.M., Ford L.G., Lippman S.M., Crawford E.D. (2004). Prevalence of prostate cancer among men with a prostate-specific antigen level ≤4.0 ng per milliliter. N. Engl. J. Med..

[6] Eeles R.A., Kote-Jarai Z., Al Olama A.A., Giles G.G., Guy M., Severi G., Muir K., Hopper J.L., Henderson B.E., Haiman C.A. (2009). Identification of seven new prostate cancer susceptibility loci through a genome-wide association study. Nat. Genet..

[7] Schumacher F.R., Berndt S.I., Siddiq A., Jacobs K.B., Wang Z., Lindstrom S., Stevens V.L., Chen C., Mondul A.M., Travis R.C. (2011). Genome-wide association study identifies new prostate cancer susceptibility loci. Hum. Mol. Genet..

[8] Haiman C.A., Chen G.K., Blot W.J., Strom S.S., Berndt S.I., Kittles R.A., Rybicki B.A., Isaacs W.B., Ingles S.A., Stanford J.L. (2011). Genome-wide association study of prostate cancer in men of African ancestry identifies a susceptibility locus at 17q21. Nat. Genet..

[9] Kote-Jarai Z., Olama A.A., Giles G.G., Severi G., Schleutker J., Weischer M., Campa D., Riboli E., Key T., Gronberg H. (2011). Seven prostate cancer susceptibility loci identified by a multi-stage genome-wide association study. Nat. Genet..

[10] Schumacher F.R., Al Olama A.A., Berndt S.I., Benlloch S., Ahmed M., Saunders E.J., Dadaev T., Leongamornlert D., Anokian E., Cieza-Borrella C. (2018). Association analyses of more than 140,000 men identify 63 new prostate cancer susceptibility loci. Nat. Genet..

[11] Siegel R.L., Miller K.D., Jemal A. (2018). Cancer statistics, 2018. CA Cancer J. Clin..

[12] Fakhrejahani F., Madan R.A., Dahut W.L. (2017). Management Options for Biochemically Recurrent Prostate Cancer. Curr. Treat. Options Oncol..

[13] Mahon K.L., Henshall S.M., Sutherland R.L., Horvath L.G. (2011). Pathways of chemotherapy resistance in castration-resistant prostate cancer. Endocr. Relat. Cancer.

[14] Galazi M., Rodriguez-Vida A., Ng T., Mason M., Chowdhury S. (2014). Precision medicine for prostate cancer. Expert Rev. Anticancer Ther..

[15] Fraser M., Sabelnykova V.Y., Yamaguchi T.N., Heisler L.E., Livingstone J., Huang V., Shiah Y.J., Yousif F., Lin X., Masella A.P. (2017). Genomic hallmarks of localized, non-indolent prostate cancer. Nature.

[16] Robinson D., Van Allen E.M., Wu Y.M., Schultz N., Lonigro R.J., Mosquera J.M., Montgomery B., Taplin M.E., Pritchard C.C., Attard G. (2015). Integrative clinical genomics of advanced prostate cancer. Cell.

[17] Rubin M.A., Demichelis F. (2018). The Genomics of Prostate Cancer: Emerging understanding with technologic advances. Mod. Pathol..

[18] The Cancer Genome Atlas Research Network (2015). The Molecular Taxonomy of Primary Prostate Cancer. Cell.

[19] Hieronymus H., Schultz N., Gopalan A., Carver B.S., Chang M.T., Xiao Y., Heguy A., Huberman K., Bernstein M., Assel M. (2014). Copy number alteration burden predicts prostate cancer relapse. Proc. Natl. Acad. Sci. USA.

[20] Bielski C.M., Zehir A., Penson A.V., Donoghue M.T.A., Chatila W., Armenia J., Chang M.T., Schram A.M., Jonsson P., Bandlamudi C. (2018). Genome doubling shapes the evolution and prognosis of advanced cancers. Nat. Genet..

[21] Armenia J., Wankowicz S.A.M., Liu D., Gao J., Kundra R., Reznik E., Chatila W.K., Chakravarty D., Han G.C., Coleman I. (2018). The long tail of oncogenic drivers in prostate cancer. Nat. Genet..

[22] Attard G., Parker C., Eeles R.A., Schröder F., Tomlins S.A., Tannock I., Drake C.G., de Bono J.S. (2016). Prostate cancer. Lancet.

[23] Weischenfeldt J., Simon R., Feuerbach L., Schlangen K., Weichenhan D., Minner S., Wuttig D., Warnatz H.J., Stehr H., Rausch T. (2013). Integrative genomic analyses reveal an androgen-driven somatic alteration landscape in early-onset prostate cancer. Cancer Cell.

[24] Lin C., Yang L., Tanasa B., Hutt K., Ju B.G., Ohgi K., Zhang J., Rose D.W., Fu X.D., Glass C.K. (2009). Nuclear receptor-induced chromosomal proximity and DNA breaks underlie specific translocations in cancer. Cell.

[25] Carver B.S., Tran J., Gopalan A., Chen Z., Shaikh S., Carracedo A., Alimonti A., Nardella C., Varmeh S., Scardino P.T. (2009). Aberrant ERG expression cooperates with loss of PTEN to promote cancer progression in the prostate. Nat. Genet..

[26] King J.C., Xu J., Wongvipat J., Hieronymus H., Carver B.S., Leung D.H., Taylor B.S., Sander C., Cardiff R.D., Couto S.S. (2009). Cooperativity of TMPRSS2-ERG with PI3-kinase pathway activation in prostate oncogenesis. Nat. Genet..

[27] Rickman D.S., Chen Y.B., Banerjee S., Pan Y., Yu J., Vuong T., Perner S., Lafargue C.J., Mertz K.D., Setlur S.R. (2010). ERG cooperates with androgen receptor in regulating trefoil factor 3 in prostate cancer disease progression. Neoplasia.

[28] Yu J., Yu J., Mani R.S., Cao Q., Brenner C.J., Cao X., Wang X., Wu L., Li J., Hu M. (2010). An integrated network of androgen receptor, polycomb, and TMPRSS2-ERG gene fusions in prostate cancer progression. Cancer Cell.

[29] Ratz L., Laible M., Kacprzyk L.A., Wittig-Blaich S.M., Tolstov Y., Duensing S., Altevogt P., Klauck S.M., Sultmann H. (2017). TMPRSS2:ERG gene fusion variants induce TGF-beta signaling and epithelial to mesenchymal transition in human prostate cancer cells. Oncotarget.

[30] Tomlins S.A., Laxman B., Varambally S., Cao X., Yu J., Helgeson B.E., Cao Q., Prensner J.R., Rubin M.A., Shah R.B. (2008). Role of the TMPRSS2-ERG gene fusion in prostate cancer. Neoplasia.

[31] Kron K.J., Murison A., Zhou S., Huang V., Yamaguchi T.N., Shiah Y.J., Fraser M., van der Kwast T., Boutros P.C., Bristow R.G. (2017). TMPRSS2-ERG fusion co-opts master transcription factors and activates NOTCH signaling in primary prostate cancer. Nat. Genet..

[32] Rickman D.S., Soong T.D., Moss B., Mosquera J.M., Dlabal J., Terry S., MacDonald T.Y., Tripodi J., Bunting K., Najfeld V. (2012). Oncogene-mediated alterations in chromatin conformation. Proc. Natl. Acad. Sci. USA.

[33] Demichelis F., Fall K., Perner S., Andren O., Schmidt F., Setlur S.R., Hoshida Y., Mosquera J.M., Pawitan Y., Lee C. (2007). TMPRSS2:ERG gene fusion associated with lethal prostate cancer in a watchful waiting cohort. Oncogene.

[34] Attard G., Clark J., Ambroisine L., Fisher G., Kovacs G., Flohr P., Berney D., Foster C.S., Fletcher A., Gerald W.L. (2008). Duplication of the fusion of TMPRSS2 to ERG sequences identifies fatal human prostate cancer. Oncogene.

[35] Tomlins S.A., Aubin S.M., Siddiqui J., Lonigro R.J., Sefton-Miller L., Miick S., Williamsen S., Hodge P., Meinke J., Blase A. (2011). Urine TMPRSS2:ERG fusion transcript stratifies prostate cancer risk in men with elevated serum PSA. Sci. Transl. Med..

[36] Gopalan A., Leversha M.A., Satagopan J.M., Zhou Q., Al-Ahmadie H.A., Fine S.W., Eastham J.A., Scardino P.T., Scher H.I., Tickoo S.K. (2009). TMPRSS2-ERG gene fusion is not associated with outcome in patients treated by prostatectomy. Cancer Res..

[37] Fine S.W., Gopalan A., Leversha M.A., Al-Ahmadie H.A., Tickoo S.K., Zhou Q., Satagopan J.M., Scardino P.T., Gerald W.L., Reuter V.E. (2010). TMPRSS2-ERG gene fusion is associated with low Gleason scores and not with high-grade morphological features. Mod. Pathol..

[38] McKiernan J., Donovan M.J., O’Neill V., Bentink S., Noerholm M., Belzer S., Skog J., Kattan M.W., Partin A., Andriole G. (2016). A Novel Urine Exosome Gene Expression Assay to Predict High-grade Prostate Cancer at Initial Biopsy. JAMA Oncol..

[39] Donovan M., Torkler P., Skog J., Noerholm M., Carroll P. (2017). Performance of a validated urine exosome gene expression assay to predict high-grade prostate cancer utilizing the International Society of Urological Pathology (ISUP) 2014 grading system. J. Clin. Oncol..

[40] Cornu J.N., Cancel-Tassin G., Egrot C., Gaffory C., Haab F., Cussenot O. (2013). Urine TMPRSS2:ERG fusion transcript integrated with PCA3 score, genotyping, and biological features are correlated to the results of prostatic biopsies in men at risk of prostate cancer. Prostate.

[41] Leyten G.H., Hessels D., Jannink S.A., Smit F.P., de Jong H., Cornel E.B., de Reijke T.M., Vergunst H., Kil P., Knipscheer B.C. (2014). Prospective multicentre evaluation of PCA3 and TMPRSS2-ERG gene fusions as diagnostic and prognostic urinary biomarkers for prostate cancer. Eur. Urol..

[42] Kulda V., Topolcan O., Kucera R., Kripnerova M., Srbecka K., Hora M., Hes O., Klecka J., Babuska V., Rousarova M. (2016). Prognostic Significance of TMPRSS2-ERG Fusion Gene in Prostate Cancer. Anticancer Res..

[43] Boysen G., Barbieri C.E., Prandi D., Blattner M., Chae S.S., Dahija A., Nataraj S., Huang D., Marotz C., Xu L. (2015). SPOP mutation leads to genomic instability in prostate cancer. Elife.

[44] Blattner M., Liu D., Robinson B.D., Huang D., Poliakov A., Gao D., Nataraj S., Deonarine L.D., Augello M.A., Sailer V. (2017). SPOP Mutation Drives Prostate Tumorigenesis In Vivo through Coordinate Regulation of PI3K/mTOR and AR Signaling. Cancer Cell.

[45] Jin H.J., Zhao J.C., Ogden I., Bergan R.C., Yu J. (2013). Androgen receptor-independent function of FoxA1 in prostate cancer metastasis. Cancer Res..

[46] Yang Y.A., Yu J. (2015). Current perspectives on FOXA1 regulation of androgen receptor signaling and prostate cancer. Genes Dis..

[47] Prensner J.R., Chinnaiyan A.M. (2011). Metabolism unhinged: IDH mutations in cancer. Nat. Med..

[48] Bunse L., Pusch S., Bunse T., Sahm F., Sanghvi K., Friedrich M., Alansary D., Sonner J.K., Green E., Deumelandt K. (2018). Suppression of antitumor T cell immunity by the oncometabolite (R)-2-hydroxyglutarate. Nat. Med..

[49] Yen K.E., Bittinger M.A., Su S.M., Fantin V.R. (2010). Cancer-associated IDH mutations: Biomarker and therapeutic opportunities. Oncogene.

[50] Miller J.J., Shih H.A., Andronesi O.C., Cahill D.P. (2017). Isocitrate dehydrogenase-mutant glioma: Evolving clinical and therapeutic implications. Cancer.

[51] Ghiam A.F., Cairns R.A., Thoms J., Dal Pra A., Ahmed O., Meng A., Mak T.W., Bristow R.G. (2012). IDH mutation status in prostate cancer. Oncogene.

[52] Barbieri C.E., Bangma C.H., Bjartell A., Catto J.W., Culig Z., Gronberg H., Luo J., Visakorpi T., Rubin M.A. (2013). The mutational landscape of prostate cancer. Eur. Urol..

[53] Bowyer S.E., Rao A.D., Lyle M., Sandhu S., Long G.V., McArthur G.A., Raleigh J.M., Hicks R.J., Millward M. (2014). Activity of trametinib in K601E and L597Q BRAF mutation-positive metastatic melanoma. Melanoma Res..

[54] Cheng Y., Tian H. (2017). Current Development Status of MEK Inhibitors. Molecules.

[55] Mateo J., Boysen G., Barbieri C.E., Bryant H.E., Castro E., Nelson P.S., Olmos D., Pritchard C.C., Rubin M.A., de Bono J.S. (2017). DNA Repair in Prostate Cancer: Biology and Clinical Implications. Eur. Urol..

[56] Weight C.J., Narayan V.M., Smith D., Kim S.P., Karnes R.J. (2017). The Effects of Population-based Prostate-specific Antigen Screening Beginning at Age 40. Urology.

[57] Grossman D.C., Curry S.J., Owens D.K., Bibbins-Domingo K., Caughey A.B., Davidson K.W., Doubeni C.A., Ebell M., Epling J.W., Kemper A.R. (2018). Screening for Prostate Cancer: US Preventive Services Task Force Recommendation Statement. JAMA.

[58] Sathianathen N.J., Konety B.R., Crook J., Saad F., Lawrentschuk N. (2018). Landmarks in prostate cancer. Nat. Rev. Urol..

[59] Mottet N., Bellmunt J., Bolla M., Briers E., Cumberbatch M.G., De Santis M., Fossati N., Gross T., Henry A.M., Joniau S. (2017). EAU-ESTRO-SIOG Guidelines on Prostate Cancer. Part 1: Screening, Diagnosis, and Local Treatment with Curative Intent. Eur. Urol..

[60] Bussemakers M.J., van Bokhoven A., Verhaegh G.W., Smit F.P., Karthaus H.F., Schalken J.A., Debruyne F.M., Ru N., Isaacs W.B. (1999). DD3: A new prostate-specific gene, highly overexpressed in prostate cancer. Cancer Res..

[61] Wei J.T., Feng Z., Partin A.W., Brown E., Thompson I., Sokoll L., Chan D.W., Lotan Y., Kibel A.S., Busby J.E. (2014). Can urinary PCA3 supplement PSA in the early detection of prostate cancer?. J. Clin. Oncol..

[62] Salami S.S., Schmidt F., Laxman B., Regan M.M., Rickman D.S., Scherr D., Bueti G., Siddiqui J., Tomlins S.A., Wei J.T. (2013). Combining urinary detection of TMPRSS2:ERG and PCA3 with serum PSA to predict diagnosis of prostate cancer. Urol. Oncol..

[63] Van Neste L., Hendriks R.J., Dijkstra S., Trooskens G., Cornel E.B., Jannink S.A., de Jong H., Hessels D., Smit F.P., Melchers W.J. (2016). Detection of High-grade Prostate Cancer Using a Urinary Molecular Biomarker-Based Risk Score. Eur. Urol..

[64] Zappala S.M., Scardino P.T., Okrongly D., Linder V., Dong Y. (2017). Clinical performance of the 4Kscore Test to predict high-grade prostate cancer at biopsy: A meta-analysis of us and European clinical validation study results. Rev. Urol..

[65] White J., Shenoy B.V., Tutrone R.F., Karsh L.I., Saltzstein D.R., Harmon W.J., Broyles D.L., Roddy T.E., Lofaro L.R., Paoli C.J. (2018). Clinical utility of the Prostate Health Index (phi) for biopsy decision management in a large group urology practice setting. Prostate Cancer Prostatic Dis..

[66] Van Neste L., Herman J.G., Otto G., Bigley J.W., Epstein J.I., Van Criekinge W. (2012). The epigenetic promise for prostate cancer diagnosis. Prostate.

[67] Mehrotra J., Varde S., Wang H., Chiu H., Vargo J., Gray K., Nagle R.B., Neri J.R., Mazumder A. (2008). Quantitative, spatial resolution of the epigenetic field effect in prostate cancer. Prostate.

[68] Carroll P.R., Parsons J.K., Andriole G., Bahnson R.R., Castle E.P., Catalona W.J., Dahl D.M., Davis J.W., Epstein J.I., Etzioni R.B. (2016). NCCN Guidelines Insights: Prostate Cancer Early Detection, Version 2.2016. J. Natl. Compr. Canc. Netw..

[69] NCCN Guidelines Version 2.2018 Prostate Cancer Early Detection. https://www.nccn.org/professionals/physician_gls/PDF/prostate_detection.pdf.

[70] McDermed J.E., Sanders R., Fait S., Klem R.E., Sarno M.J., Adams T.H., Diamandis E.P. (2012). Nucleic acid detection immunoassay for prostate-specific antigen based on immuno-PCR methodology. Clin. Chem..

[71] Moul J.W., Lilja H., Semmes O.J., Lance R.S., Vessella R.L., Fleisher M., Mazzola C., Sarno M.J., Stevens B., Klem R.E. (2012). NADiA ProsVue prostate-specific antigen slope is an independent prognostic marker for identifying men at reduced risk of clinical recurrence of prostate cancer after radical prostatectomy. Urology.

[72] Moul J.W., Sarno M.J., McDermed J.E., Triebell M.T., Reynolds M.A. (2014). NADiA ProsVue prostate-specific antigen slope, CAPRA-S, and prostate cancer--specific survival after radical prostatectomy. Urology.

[73] Carroll P.H., Mohler J.L. (2018). NCCN Guidelines Updates: Prostate Cancer and Prostate Cancer Early Detection. J. Natl. Compr. Canc. Netw..

[74] NCCN Guidelines Version 2.2018 Prostate Cancer. https://www.nccn.org/professionals/physician_gls/pdf/prostate.pdf.

[75] Erho N., Crisan A., Vergara I.A., Mitra A.P., Ghadessi M., Buerki C., Bergstralh E.J., Kollmeyer T., Fink S., Haddad Z. (2013). Discovery and validation of a prostate cancer genomic classifier that predicts early metastasis following radical prostatectomy. PLoS ONE.

[76] Cullen J., Rosner I.L., Brand T.C., Zhang N., Tsiatis A.C., Moncur J., Ali A., Chen Y., Knezevic D., Maddala T. (2015). A Biopsy-based 17-gene Genomic Prostate Score Predicts Recurrence After Radical Prostatectomy and Adverse Surgical Pathology in a Racially Diverse Population of Men with Clinically Low- and Intermediate-risk Prostate Cancer. Eur. Urol..

[77] Klein E.A., Cooperberg M.R., Magi-Galluzzi C., Simko J.P., Falzarano S.M., Maddala T., Chan J.M., Li J., Cowan J.E., Tsiatis A.C. (2014). A 17-gene assay to predict prostate cancer aggressiveness in the context of Gleason grade heterogeneity, tumor multifocality, and biopsy undersampling. Eur. Urol..

[78] Cuzick J., Swanson G.P., Fisher G., Brothman A.R., Berney D.M., Reid J.E., Mesher D., Speights V.O., Stankiewicz E., Foster C.S. (2011). Prognostic value of an RNA expression signature derived from cell cycle proliferation genes in patients with prostate cancer: A retrospective study. Lancet Oncol..

[79] Sommariva S., Tarricone R., Lazzeri M., Ricciardi W., Montorsi F. (2016). Prognostic Value of the Cell Cycle Progression Score in Patients with Prostate Cancer: A Systematic Review and Meta-analysis. Eur. Urol..

[80] Shipitsin M., Small C., Choudhury S., Giladi E., Friedlander S., Nardone J., Hussain S., Hurley A.D., Ernst C., Huang Y.E. (2014). Identification of proteomic biomarkers predicting prostate cancer aggressiveness and lethality despite biopsy-sampling error. Br. J. Cancer.

[81] Shipitsin M., Small C., Giladi E., Siddiqui S., Choudhury S., Hussain S., Huang Y.E., Chang H., Rimm D.L., Berman D.M. (2014). Automated quantitative multiplex immunofluorescence in situ imaging identifies phospho-S6 and phospho-PRAS40 as predictive protein biomarkers for prostate cancer lethality. Proteome Sci..

[82] Tomlins S.A., Day J.R., Lonigro R.J., Hovelson D.H., Siddiqui J., Kunju L.P., Dunn R.L., Meyer S., Hodge P., Groskopf J. (2016). Urine TMPRSS2:ERG Plus PCA3 for Individualized Prostate Cancer Risk Assessment. Eur. Urol..

[83] Sanda M.G., Feng Z., Howard D.H., Tomlins S.A., Sokoll L.J., Chan D.W., Regan M.M., Groskopf J., Chipman J., Patil D.H. (2017). Association Between Combined TMPRSS2:ERG and PCA3 RNA Urinary Testing and Detection of Aggressive Prostate Cancer. JAMA Oncol..

[84] Parekh D.J., Punnen S., Sjoberg D.D., Asroff S.W., Bailen J.L., Cochran J.S., Concepcion R., David R.D., Deck K.B., Dumbadze I. (2015). A multi-institutional prospective trial in the USA confirms that the 4Kscore accurately identifies men with high-grade prostate cancer. Eur. Urol..

[85] Punnen S., Freedland S.J., Polascik T.J., Loeb S., Risk M.C., Savage S., Mathur S.C., Uchio E., Dong Y., Silberstein J.L. (2017). A Multi-Institutional Prospective Trial Confirms Noninvasive Blood Test Maintains Predictive Value in African American Men. J. Urol..

[86] Gupta A., Roobol M.J., Savage C.J., Peltola M., Pettersson K., Scardino P.T., Vickers A.J., Schroder F.H., Lilja H. (2010). A four-kallikrein panel for the prediction of repeat prostate biopsy: Data from the European Randomized Study of Prostate Cancer screening in Rotterdam, Netherlands. Br. J. Cancer.

[87] Partin A.W., Van Neste L., Klein E.A., Marks L.S., Gee J.R., Troyer D.A., Rieger-Christ K., Jones J.S., Magi-Galluzzi C., Mangold L.A. (2014). Clinical validation of an epigenetic assay to predict negative histopathological results in repeat prostate biopsies. J. Urol..

[88] Stewart G.D., Van Neste L., Delvenne P., Delree P., Delga A., McNeill S.A., O’Donnell M., Clark J., Van Criekinge W., Bigley J. (2013). Clinical utility of an epigenetic assay to detect occult prostate cancer in histopathologically negative biopsies: Results of the MATLOC study. J. Urol..

[89] Tosoian J.J., Druskin S.C., Andreas D., Mullane P., Chappidi M., Joo S., Ghabili K., Agostino J., Macura K.J., Carter H.B. (2017). Use of the Prostate Health Index for detection of prostate cancer: Results from a large academic practice. Prostate Cancer Prostatic Dis..

[90] Scattoni V., Lazzeri M., Lughezzani G., De Luca S., Passera R., Bollito E., Randone D., Abdollah F., Capitanio U., Larcher A. (2013). Head-to-head comparison of prostate health index and urinary PCA3 for predicting cancer at initial or repeat biopsy. J. Urol..

[91] Clinton T.N., Bagrodia A., Lotan Y., Margulis V., Raj G.V., Woldu S.L. (2017). Tissue-based biomarkers in prostate cancer. Expert Rev. Precis. Med. Drug Dev..

[92] Blume-Jensen P., Berman D.M., Rimm D.L., Shipitsin M., Putzi M., Nifong T.P., Small C., Choudhury S., Capela T., Coupal L. (2015). Development and clinical validation of an in situ biopsy-based multimarker assay for risk stratification in prostate cancer. Clin. Cancer Res..

[93] Sharma P., Zargar-Shoshtari K., Pow-Sang J.M. (2016). Biomarkers for prostate cancer: Present challenges and future opportunities. Future Sci. OA.

[94] Strand S.H., Orntoft T.F., Sorensen K.D. (2014). Prognostic DNA methylation markers for prostate cancer. Int. J. Mol. Sci..

[95] Mohler J.L., Armstrong A.J., Bahnson R.R., D’Amico A.V., Davis B.J., Eastham J.A., Enke C.A., Farrington T.A., Higano C.S., Horwitz E.M. (2016). Prostate Cancer, Version 1.2016. J. Natl. Compr. Canc. Netw..

[96] Bill-Axelson A., Holmberg L., Filen F., Ruutu M., Garmo H., Busch C., Nordling S., Haggman M., Andersson S.O., Bratell S. (2008). Radical prostatectomy versus watchful waiting in localized prostate cancer: The Scandinavian prostate cancer group-4 randomized trial. J. Natl. Cancer Inst..

[97] Bill-Axelson A., Holmberg L., Garmo H., Rider J.R., Taari K., Busch C., Nordling S., Haggman M., Andersson S.O., Spangberg A. (2014). Radical prostatectomy or watchful waiting in early prostate cancer. N. Engl. J. Med..

[98] Ahmed H.U., Hindley R.G., Dickinson L., Freeman A., Kirkham A.P., Sahu M., Scott R., Allen C., Van der Meulen J., Emberton M. (2012). Focal therapy for localised unifocal and multifocal prostate cancer: A prospective development study. Lancet Oncol..

[99] Messing E.M., Manola J., Yao J., Kiernan M., Crawford D., Wilding G., di’SantAgnese P.A., Trump D. (2006). On behalf of the Eastern Cooperative Oncology Group study EST 3886. Immediate versus deferred androgen deprivation treatment in patients with node-positive prostate cancer after radical prostatectomy and pelvic lymphadenectomy. Lancet Oncol..

[100] Safety and Efficacy Study of Enzalutamide Plus Leuprolide in Patients With Nonmetastatic Prostate Cancer (EMBARK). https://ClinicalTrials.gov/show/NCT02319837.

[101] Bubendorf L., Schopfer A., Wagner U., Sauter G., Moch H., Willi N., Gasser T.C., Mihatsch M.J. (2000). Metastatic patterns of prostate cancer: An autopsy study of 1589 patients. Hum. Pathol..

[102] Sartor O., de Bono J.S. (2018). Metastatic Prostate Cancer. N. Engl. J. Med..

[103] Montero A., Fossella F., Hortobagyi G., Valero V. (2005). Docetaxel for treatment of solid tumours: A systematic review of clinical data. Lancet Oncol..

[104] Parker C., Nilsson S., Heinrich D., Helle S.I., O’Sullivan J.M., Fossa S.D., Chodacki A., Wiechno P., Logue J., Seke M. (2013). Alpha emitter radium-223 and survival in metastatic prostate cancer. N. Engl. J. Med..

[105] Kantoff P.W., Higano C.S., Shore N.D., Berger E.R., Small E.J., Penson D.F., Redfern C.H., Ferrari A.C., Dreicer R., Sims R.B. (2010). Sipuleucel-T immunotherapy for castration-resistant prostate cancer. N. Engl. J. Med..

[106] Saad F., McKiernan J., Eastham J. (2006). Rationale for zoledronic acid therapy in men with hormone-sensitive prostate cancer with or without bone metastasis. Urol. Oncol..

[107] Fizazi K., Carducci M., Smith M., Damiao R., Brown J., Karsh L., Milecki P., Shore N., Rader M., Wang H. (2011). Denosumab versus zoledronic acid for treatment of bone metastases in men with castration-resistant prostate cancer: A randomised, double-blind study. Lancet.

[108] Brown J.E., Cleeland C.S., Fallowfield L.J., Patrick D.L., Fizazi K., Smith M.R., Maroto J.P., Michel M.S., Feng A., Goessl C. (2011). Pain outcomes in patients with bone metastases from castrate-resistant prostate cancer: Results from a phase 3 trials of denosumab vs. zoledronic acid. Eur. Urol. Suppl..

[109] Patrick D., Cleeland C.S., Fallowfield L., Smith M.R., Klotz L., Oudard S., Marx G.M., Wei R., Ohrling K., Qian Y. (2014). Denosumab or zoledronic acid (ZA) therapy on pain interference and cancer-specific quality of life (CSQoL) in patients with castrate-resistant prostate cancer (CRPC) and bone metastases (BM). J. Clin. Oncol..

[110] Hegemann M., Bedke J., Stenzl A., Todenhofer T. (2017). Denosumab treatment in the management of patients with advanced prostate cancer: Clinical evidence and experience. Ther. Adv. Urol..

[111] Taylor B.S., Schultz N., Hieronymus H., Gopalan A., Xiao Y., Carver B.S., Arora V.K., Kaushik P., Cerami E., Reva B. (2010). Integrative genomic profiling of human prostate cancer. Cancer Cell.

[112] Viswanathan S.R., Ha G., Hoff A.M., Wala J.A., Carrot-Zhang J., Whelan C.W., Haradhvala N.J., Freeman S.S., Reed S.C., Rhoades J. (2018). Structural Alterations Driving Castration-Resistant Prostate Cancer Revealed by Linked-Read Genome Sequencing. Cell.

[113] Takeda D.Y., Spisak S., Seo J.H., Bell C., O’Connor E., Korthauer K., Ribli D., Csabai I., Solymosi N., Szallasi Z. (2018). A Somatically Acquired Enhancer of the Androgen Receptor Is a Noncoding Driver in Advanced Prostate Cancer. Cell.

[114] Quigley D.A., Dang H.X., Zhao S.G., Lloyd P., Aggarwal R., Alumkal J.J., Foye A., Kothari V., Perry M.D., Bailey A.M. (2018). Genomic Hallmarks and Structural Variation in Metastatic Prostate Cancer. Cell.

[115] Gao P., Xia J.-H., Sipeky C., Dong X.-M., Zhang Q., Yang Y., Zhang P., Cruz S.P., Zhang K., Zhu J. (2018). Biology and Clinical Implications of the 19q13 Aggressive Prostate Cancer Susceptibility Locus. Cell.

[116] Grasso C.S., Wu Y.M., Robinson D.R., Cao X., Dhanasekaran S.M., Khan A.P., Quist M.J., Jing X., Lonigro R.J., Brenner J.C. (2012). The mutational landscape of lethal castration-resistant prostate cancer. Nature.

[117] Ku S.Y., Rosario S., Wang Y., Mu P., Seshadri M., Goodrich Z.W., Goodrich M.M., Labbe D.P., Gomez E.C., Wang J. (2017). Rb1 and Trp53 cooperate to suppress prostate cancer lineage plasticity, metastasis, and antiandrogen resistance. Science.

[118] Malhotra A., Shibata Y., Hall I.M., Dutta A. (2013). Chromosomal structural variations during progression of a prostate epithelial cell line to a malignant metastatic state inactivate the NF2, NIPSNAP1, UGT2B17, and LPIN2 genes. Cancer Biol. Ther..

[119] Jaratlerdsiri W., Chan E.K.F., Petersen D.C., Yang C., Croucher P.I., Bornman M.S.R., Sheth P., Hayes V.M. (2017). Next generation mapping reveals novel large genomic rearrangements in prostate cancer. Oncotarget.

[120] Yoshimoto M., Cunha I.W., Coudry R.A., Fonseca F.P., Torres C.H., Soares F.A., Squire J.A. (2007). FISH analysis of 107 prostate cancers shows that PTEN genomic deletion is associated with poor clinical outcome. Br. J. Cancer.

[121] Dillon L.M., Miller T.W. (2014). Therapeutic targeting of cancers with loss of PTEN function. Curr. Drug Targets.

[122] Mendes-Pereira A.M., Martin S.A., Brough R., McCarthy A., Taylor J.R., Kim J.S., Waldman T., Lord C.J., Ashworth A. (2009). Synthetic lethal targeting of PTEN mutant cells with PARP inhibitors. EMBO Mol. Med..

[123] De Bono J.S., De Giorgi U., Massard C., Bracarda S., Nava Rodrigues D., Kocak I., Font A., Arija J.A., Shih K., Radavoi G.D. (2016). PTEN loss as a predictive biomarker for the Akt inhibitor ipatasertib combined with abiraterone acetate in patients with metastatic castration-resistant prostate cancer (mCRPC). Ann. Oncol..

[124] Sweeney C.J., Chen Y.H., Carducci M., Liu G., Jarrard D.F., Eisenberger M., Wong Y.N., Hahn N., Kohli M., Cooney M.M. (2015). Chemohormonal Therapy in Metastatic Hormone-Sensitive Prostate Cancer. N. Engl. J. Med..

[125] James N.D., Sydes M.R., Clarke N.W., Mason M.D., Dearnaley D.P., Spears M.R., Ritchie A.W., Parker C.C., Russell J.M., Attard G. (2016). Addition of docetaxel, zoledronic acid, or both to first-line long-term hormone therapy in prostate cancer (STAMPEDE): Survival results from an adaptive, multiarm, multistage, platform randomised controlled trial. Lancet.

[126] Fizazi K., Tran N., Fein L., Matsubara N., Rodriguez-Antolin A., Alekseev B.Y., Ozguroglu M., Ye D., Feyerabend S., Protheroe A. (2017). Abiraterone plus Prednisone in Metastatic, Castration-Sensitive Prostate Cancer. N. Engl. J. Med..

[127] James N.D., de Bono J.S., Spears M.R., Clarke N.W., Mason M.D., Dearnaley D.P., Ritchie A.W.S., Amos C.L., Gilson C., Jones R.J. (2017). Abiraterone for Prostate Cancer Not Previously Treated with Hormone Therapy. N. Engl. J. Med..

[128] De Bono J.S., Logothetis C.J., Molina A., Fizazi K., North S., Chu L., Chi K.N., Jones R.J., Goodman O.B., Saad F. (2011). Abiraterone and increased survival in metastatic prostate cancer. N. Engl. J. Med..

[129] Ryan C.J., Smith M.R., de Bono J.S., Molina A., Logothetis C.J., de Souza P., Fizazi K., Mainwaring P., Piulats J.M., Ng S. (2013). Abiraterone in metastatic prostate cancer without previous chemotherapy. N. Engl. J. Med..

[130] Scher H.I., Fizazi K., Saad F., Taplin M.E., Sternberg C.N., Miller K., de Wit R., Mulders P., Chi K.N., Shore N.D. (2012). Increased survival with enzalutamide in prostate cancer after chemotherapy. N. Engl. J. Med..

[131] Beer T.M., Armstrong A.J., Rathkopf D.E., Loriot Y., Sternberg C.N., Higano C.S., Iversen P., Bhattacharya S., Carles J., Chowdhury S. (2014). Enzalutamide in metastatic prostate cancer before chemotherapy. N. Engl. J. Med..

[132] Smith M.R., Yu M.K., Small E.J. (2018). Apalutamide and Metastasis-free Survival in Prostate Cancer. N. Engl. J. Med..

[133] Chen C.D., Welsbie D.S., Tran C., Baek S.H., Chen R., Vessella R., Rosenfeld M.G., Sawyers C.L. (2004). Molecular determinants of resistance to antiandrogen therapy. Nat. Med..

[134] Li H., Wang Z., Xiao W., Yan L., Guan W., Hu Z., Wu L., Huang Q., Wang J., Xu H. (2018). Androgen-receptor splice variant-7-positive prostate cancer: A novel molecular subtype with markedly worse androgen-deprivation therapy outcomes in newly diagnosed patients. Mod. Pathol..

[135] Antonarakis E.S., Lu C., Wang H., Luber B., Nakazawa M., Roeser J.C., Chen Y., Mohammad T.A., Chen Y., Fedor H.L. (2014). AR-V7 and resistance to enzalutamide and abiraterone in prostate cancer. N. Engl. J. Med..

[136] Papazoglou D., Wannesson L., Berthold D., Cathomas R., Gillessen S., Rothermundt C., Hasler L., Winterhalder R., Barth A., Mingrone W. (2017). Enzalutamide in Patients With Castration-Resistant Prostate Cancer Progressing After Docetaxel: Retrospective Analysis of the Swiss Enzalutamide Named Patient Program. Clin. Genitourin Cancer.

[137] De Felice F., Tombolini V., Marampon F., Musella A., Marchetti C. (2017). Defective DNA repair mechanisms in prostate cancer: Impact of olaparib. Drug Des. Devel. Ther..

[138] Mateo J., Carreira S., Sandhu S., Miranda S., Mossop H., Perez-Lopez R., Nava Rodrigues D., Robinson D., Omlin A., Tunariu N. (2015). DNA-Repair Defects and Olaparib in Metastatic Prostate Cancer. N. Engl. J. Med..

[139] Ph II Study to Evaluate Olaparib With Abiraterone in Treating Metastatic Castration Resistant Prostate Cancer. https://ClinicalTrials.gov/show/NCT01972217.

[140] Phase I/II Study of the Anti-Programmed Death Ligand-1 Antibody MEDI4736 in Combination With Olaparib and/or Cediranib for Advanced Solid Tumors and Advanced or Recurrent Ovarian, Triple Negative Breast, Lung, Prostate and Colorectal Cancers. https://ClinicalTrials.gov/show/NCT02484404.

[141] TOPARP: A Phase II Trial of Olaparib in Patients With Advanced Castration Resistant Prostate Cancer. https://ClinicalTrials.gov/show/NCT01682772.

[142] Jamaspishvili T., Berman D.M., Ross A.E., Scher H.I., De Marzo A.M., Squire J.A., Lotan T.L. (2018). Clinical implications of PTEN loss in prostate cancer. Nat. Rev. Urol..

[143] Brenner J.C., Ateeq B., Li Y., Yocum A.K., Cao Q., Asangani I.A., Patel S., Wang X., Liang H., Yu J. (2011). Mechanistic rationale for inhibition of poly(ADP-ribose) polymerase in ETS gene fusion-positive prostate cancer. Cancer Cell.

[144] Hussain M., Daignault-Newton S., Twardowski P.W., Albany C., Stein M.N., Kunju L.P., Siddiqui J., Wu Y.M., Robinson D., Lonigro R.J. (2018). Targeting Androgen Receptor and DNA Repair in Metastatic Castration-Resistant Prostate Cancer: Results From NCI 9012. J. Clin. Oncol..

[145] Chatterjee P., Choudhary G.S., Sharma A., Singh K., Heston W.D., Ciezki J., Klein E.A., Almasan A. (2013). PARP inhibition sensitizes to low dose-rate radiation TMPRSS2-ERG fusion gene-expressing and PTEN-deficient prostate cancer cells. PLoS ONE.

[146] Buonerba C., Federico P., D’Aniello C., Rescigno P., Cavaliere C., Puglia L., Ferro M., Altieri V., Perdona S., De Placido S. (2011). Phase II trial of cisplatin plus prednisone in docetaxel-refractory castration-resistant prostate cancer patients. Cancer Chemother. Pharmacol..

[147] Kentepozidis N., Soultati A., Giassas S., Vardakis N., Kalykaki A., Kotsakis A., Papadimitraki E., Pantazopoulos N., Bozionellou V., Georgoulias V. (2012). Paclitaxel in combination with carboplatin as salvage treatment in patients with castration-resistant prostate cancer: A Hellenic oncology research group multicenter phase II study. Cancer Chemother. Pharmacol..

[148] Urakami S., Igawa M., Kikuno N., Yoshino T., Kishi H., Shigeno K., Shiina H. (2002). Combination chemotherapy with paclitaxel, estramustine and carboplatin for hormone refractory prostate cancer. J. Urol..

[149] Droz J.P., Muracciole X., Mottet N., Ould Kaci M., Vannetzel J.M., Albin N., Culine S., Rodier J.M., Misset J.L., Mackenzie S. (2003). Phase II study of oxaliplatin versus oxaliplatin combined with infusional 5-fluorouracil in hormone refractory metastatic prostate cancer patients. Ann. Oncol..

[150] Culine S., El Demery M., Lamy P.J., Iborra F., Avances C., Pinguet F. (2007). Docetaxel and cisplatin in patients with metastatic androgen independent prostate cancer and circulating neuroendocrine markers. J. Urol..

[151] Papandreou C.N., Daliani D.D., Thall P.F., Tu S.M., Wang X., Reyes A., Troncoso P., Logothetis C.J. (2002). Results of a phase II study with doxorubicin, etoposide, and cisplatin in patients with fully characterized small-cell carcinoma of the prostate. J. Clin. Oncol..

[152] Miglietta L., Cannobbio L., Boccardo F. (1995). Assessment of response to carboplatin in patients with hormone-refractory prostate cancer: A critical analysis of drug activity. Anticancer Res..

[153] Sternberg C.N., Petrylak D.P., Sartor O., Witjes J.A., Demkow T., Ferrero J.M., Eymard J.C., Falcon S., Calabro F., James N. (2009). Multinational, double-blind, phase III study of prednisone and either satraplatin or placebo in patients with castrate-refractory prostate cancer progressing after prior chemotherapy: The SPARC trial. J. Clin. Oncol..

[154] Hager S., Ackermann C.J., Joerger M., Gillessen S., Omlin A. (2016). Anti-tumour activity of platinum compounds in advanced prostate cancer-a systematic literature review. Ann. Oncol..

[155] Carboplatin in Castration-resistant Prostate Cancer. https://ClinicalTrials.gov/show/NCT02311764.

[156] Docetaxel and Carboplatin in Treating Patients With Metastatic, Castration Resistant Prostate Cancer Containing Inactivated Genes in the BRCA 1/2 Pathway. https://ClinicalTrials.gov/show/NCT02598895.

[157] Hanahan D., Weinberg R.A. (2011). Hallmarks of cancer: The next generation. Cell.

[158] Pritchard C.C., Morrissey C., Kumar A., Zhang X., Smith C., Coleman I., Salipante S.J., Milbank J., Yu M., Grady W.M. (2014). Complex MSH2 and MSH6 mutations in hypermutated microsatellite unstable advanced prostate cancer. Nat. Commun..

[159] Le D.T., Uram J.N., Wang H., Bartlett B.R., Kemberling H., Eyring A.D., Skora A.D., Luber B.S., Azad N.S., Laheru D. (2015). PD-1 Blockade in Tumors with Mismatch-Repair Deficiency. N. Engl. J. Med..

[160] Xu-Monette Z.Y., Zhang M., Li J., Young K.H. (2017). PD-1/PD-L1 Blockade: Have We Found the Key to Unleash the Antitumor Immune Response?. Front. Immunol..

[161] Graff J.N., Alumkal J.J., Drake C.G., Thomas G.V., Redmond W.L., Farhad M., Cetnar J.P., Ey F.S., Bergan R.C., Slottke R. (2016). Early evidence of anti-PD-1 activity in enzalutamide-resistant prostate cancer. Oncotarget.

[162] Hansen A., Massard C., Ott P.A., Haas N., Lopez J., Ejadi S., Wallmark J., Keam B., Delord J.P., Aggarwal R. (2016). Pembrolizumab for patients with advanced prostate adenocarcinoma: Preliminary results from the KEYNOTE-028 study. Ann. Oncol..

[163] Brahmer J.R., Tykodi S.S., Chow L.Q., Hwu W.J., Topalian S.L., Hwu P., Drake C.G., Camacho L.H., Kauh J., Odunsi K. (2012). Safety and activity of anti-PD-L1 antibody in patients with advanced cancer. N. Engl. J. Med..

[164] Bishop J.L., Sio A., Angeles A., Roberts M.E., Azad A.A., Chi K.N., Zoubeidi A. (2015). PD-L1 is highly expressed in Enzalutamide resistant prostate cancer. Oncotarget.

[165] Haffner M.C., Guner G., Taheri D., Netto G.J., Palsgrove D.N., Zheng Q., Guedes L.B., Kim K., Tsai H., Esopi D.M. (2018). Comprehensive Evaluation of Programmed Death-Ligand 1 Expression in Primary and Metastatic Prostate Cancer. Am. J. Pathol..

[166] Wu Y.M., Cieslik M., Lonigro R.J., Vats P., Reimers M.A., Cao X., Ning Y., Wang L., Kunju L.P., de Sarkar N. (2018). Inactivation of CDK12 Delineates a Distinct Immunogenic Class of Advanced Prostate Cancer. Cell.

[167] Doroshow D.B., Eder J.P., LoRusso P.M. (2017). BET inhibitors: A novel epigenetic approach. Ann. Oncol..

[168] Papavassiliou K.A., Papavassiliou A.G. (2014). Bromodomains: Pockets with therapeutic potential. Trends Mol. Med..

[169] Delmore J.E., Issa G.C., Lemieux M.E., Rahl P.B., Shi J., Jacobs H.M., Kastritis E., Gilpatrick T., Paranal R.M., Qi J. (2011). BET bromodomain inhibition as a therapeutic strategy to target c-Myc. Cell.

[170] Gurel B., Iwata T., Koh C.M., Jenkins R.B., Lan F., Van Dang C., Hicks J.L., Morgan J., Cornish T.C., Sutcliffe S. (2008). Nuclear MYC protein overexpression is an early alteration in human prostate carcinogenesis. Mod. Pathol..

[171] Iwata T., Schultz D., Hicks J., Hubbard G.K., Mutton L.N., Lotan T.L., Bethel C., Lotz M.T., Yegnasubramanian S., Nelson W.G. (2010). MYC overexpression induces prostatic intraepithelial neoplasia and loss of Nkx3.1 in mouse luminal epithelial cells. PLoS ONE.

[172] Jenkins R.B., Qian J., Lieber M.M., Bostwick D.G. (1997). Detection of c-myc oncogene amplification and chromosomal anomalies in metastatic prostatic carcinoma by fluorescence in situ hybridization. Cancer Res..

[173] Asangani I.A., Dommeti V.L., Wang X., Malik R., Cieslik M., Yang R., Escara-Wilke J., Wilder-Romans K., Dhanireddy S., Engelke C. (2014). Therapeutic targeting of BET bromodomain proteins in castration-resistant prostate cancer. Nature.

[174] Asangani I.A., Wilder-Romans K., Dommeti V.L., Krishnamurthy P.M., Apel I.J., Escara-Wilke J., Plymate S.R., Navone N.M., Wang S., Feng F.Y. (2016). BET Bromodomain Inhibitors Enhance Efficacy and Disrupt Resistance to AR Antagonists in the Treatment of Prostate Cancer. Mol. Cancer Res..

[175] Dai X., Gan W., Li X., Wang S., Zhang W., Huang L., Liu S., Zhong Q., Guo J., Zhang J. (2017). Prostate cancer-associated SPOP mutations confer resistance to BET inhibitors through stabilization of BRD4. Nat. Med..

[176] Zhang P., Wang D., Zhao Y., Ren S., Gao K., Ye Z., Wang S., Pan C.W., Zhu Y., Yan Y. (2017). Intrinsic BET inhibitor resistance in SPOP-mutated prostate cancer is mediated by BET protein stabilization and AKT-mTORC1 activation. Nat. Med..

[177] A Study of ZEN003694 in Patients With Metastatic Castration-Resistant Prostate Cancer. https://ClinicalTrials.gov/show/NCT02705469.

[178] A Dose-Finding Study of OTX105/MK-8628, a Small Molecule Inhibitor of the Bromodomain and Extra-Terminal (BET) Proteins, in Adults With Selected Advanced Solid Tumors (MK-8628-003). https://ClinicalTrials.gov/show/NCT02259114.

[179] Beltran H., Rickman D.S., Park K., Chae S.S., Sboner A., MacDonald T.Y., Wang Y., Sheikh K.L., Terry S., Tagawa S.T. (2011). Molecular characterization of neuroendocrine prostate cancer and identification of new drug targets. Cancer Discov..

[180] Beltran H., Prandi D., Mosquera J.M., Benelli M., Puca L., Cyrta J., Marotz C., Giannopoulou E., Chakravarthi B.V., Varambally S. (2016). Divergent clonal evolution of castration-resistant neuroendocrine prostate cancer. Nat. Med..

[181] Epstein J.I., Amin M.B., Beltran H., Lotan T.L., Mosquera J.M., Reuter V.E., Robinson B.D., Troncoso P., Rubin M.A. (2014). Proposed morphologic classification of prostate cancer with neuroendocrine differentiation. Am. J. Surg. Pathol..

[182] Qi J., Pellecchia M., Ronai Z.A. (2010). The Siah2-HIF-FoxA2 axis in prostate cancer—new markers and therapeutic opportunities. Oncotarget.

[183] Wang H.T., Yao Y.H., Li B.G., Tang Y., Chang J.W., Zhang J. (2014). Neuroendocrine Prostate Cancer (NEPC) progressing from conventional prostatic adenocarcinoma: Factors associated with time to development of NEPC and survival from NEPC diagnosis-a systematic review and pooled analysis. J. Clin. Oncol..

[184] Gillessen S., Omlin A., Attard G., de Bono J.S., Efstathiou E., Fizazi K., Halabi S., Nelson P.S., Sartor O., Smith M.R. (2015). Management of patients with advanced prostate cancer: Recommendations of the St Gallen Advanced Prostate Cancer Consensus Conference (APCCC) 2015. Ann. Oncol..

[185] Bonkhoff H., Stein U., Remberger K. (1995). Endocrine-paracrine cell types in the prostate and prostatic adenocarcinoma are postmitotic cells. Hum. Pathol..

[186] Sauer C.G., Roemer A., Grobholz R. (2006). Genetic analysis of neuroendocrine tumor cells in prostatic carcinoma. Prostate.

[187] Lotan T.L., Gupta N.S., Wang W., Toubaji A., Haffner M.C., Chaux A., Hicks J.L., Meeker A.K., Bieberich C.J., De Marzo A.M. (2011). ERG gene rearrangements are common in prostatic small cell carcinomas. Mod. Pathol..

[188] Hansel D.E., Nakayama M., Luo J., Abukhdeir A.M., Park B.H., Bieberich C.J., Hicks J.L., Eisenberger M., Nelson W.G., Mostwin J.L. (2009). Shared TP53 gene mutation in morphologically and phenotypically distinct concurrent primary small cell neuroendocrine carcinoma and adenocarcinoma of the prostate. Prostate.

[189] Beltran H., Tagawa S.T., Park K., MacDonald T., Milowsky M.I., Mosquera J.M., Rubin M.A., Nanus D.M. (2012). Challenges in recognizing treatment-related neuroendocrine prostate cancer. J. Clin. Oncol..

[190] Wang W., Epstein J.I. (2008). Small cell carcinoma of the prostate. A morphologic and immunohistochemical study of 95 cases. Am. J. Surg. Pathol..

[191] Yuan T.C., Veeramani S., Lin F.F., Kondrikou D., Zelivianski S., Igawa T., Karan D., Batra S.K., Lin M.F. (2006). Androgen deprivation induces human prostate epithelial neuroendocrine differentiation of androgen-sensitive LNCaP cells. Endocr. Relat. Cancer.

[192] Aparicio A.M., Shen L., Tapia E.L., Lu J.F., Chen H.C., Zhang J., Wu G., Wang X., Troncoso P., Corn P. (2016). Combined Tumor Suppressor Defects Characterize Clinically Defined Aggressive Variant Prostate Cancers. Clin. Cancer Res..

[193] ProSTAR: A Study Evaluating CPI-1205 in Patients With Metastatic Castration Resistant Prostate Cancer. https://ClinicalTrials.gov/show/NCT03480646.

[194] Svensson C., Ceder J., Iglesias-Gato D., Chuan Y.C., Pang S.T., Bjartell A., Martinez R.M., Bott L., Helczynski L., Ulmert D. (2014). REST mediates androgen receptor actions on gene repression and predicts early recurrence of prostate cancer. Nucleic Acids Res..

[195] Borno S.T., Fischer A., Kerick M., Falth M., Laible M., Brase J.C., Kuner R., Dahl A., Grimm C., Sayanjali B. (2012). Genome-wide DNA methylation events in TMPRSS2-ERG fusion-negative prostate cancers implicate an EZH2-dependent mechanism with miR-26a hypermethylation. Cancer Discov..

[196] Aggarwal R., Huang J., Alumkal J.J., Zhang L., Feng F.Y., Thomas G.V., Weinstein A.S., Friedl V., Zhang C., Witte O.N. (2018). Clinical and Genomic Characterization of Treatment-Emergent Small-Cell Neuroendocrine Prostate Cancer: A Multi-institutional Prospective Study. J. Clin. Oncol..

[197] Bishop J.L., Thaper D., Vahid S., Davies A., Ketola K., Kuruma H., Jama R., Nip K.M., Angeles A., Johnson F. (2017). The Master Neural Transcription Factor BRN2 Is an Androgen Receptor-Suppressed Driver of Neuroendocrine Differentiation in Prostate Cancer. Cancer Discov..

[198] Lee J.K., Phillips J.W., Smith B.A., Park J.W., Stoyanova T., McCaffrey E.F., Baertsch R., Sokolov A., Meyerowitz J.G., Mathis C. (2016). N-Myc Drives Neuroendocrine Prostate Cancer Initiated from Human Prostate Epithelial Cells. Cancer Cell.

[199] Niu H., Manfredi M., Ecsedy J.A. (2015). Scientific Rationale Supporting the Clinical Development Strategy for the Investigational Aurora A Kinase Inhibitor Alisertib in Cancer. Front. Oncol..

[200] Kivinummi K., Urbanucci A., Leinonen K., Tammela T.L.J., Annala M., Isaacs W.B., Bova G.S., Nykter M., Visakorpi T. (2017). The expression of AURKA is androgen regulated in castration-resistant prostate cancer. Sci. Rep..

[201] Potosky A.L., Davis W.W., Hoffman R.M., Stanford J.L., Stephenson R.A., Penson D.F., Harlan L.C. (2004). Five-year outcomes after prostatectomy or radiotherapy for prostate cancer: The prostate cancer outcomes study. J. Natl. Cancer Inst..

[202] Alisertib, Abiraterone Acetate and Prednisone in Treating Patients With Hormone-Resistant Prostate Cancer. https://ClinicalTrials.gov/show/NCT01848067.

[203] Dash A., Chakraborty S., Pillai M.R., Knapp F.F. (2015). Peptide receptor radionuclide therapy: An overview. Cancer Biother. Radiopharm..

[204] Lutetium-177 (Lu177) Prostate-Specific Antigen (PSMA)-Directed EndoRadiotherapy. https://ClinicalTrials.gov/show/NCT03042312.

[205] Penney K.L., Stampfer M.J., Jahn J.L., Sinnott J.A., Flavin R., Rider J.R., Finn S., Giovannucci E., Sesso H.D., Loda M. (2013). Gleason grade progression is uncommon. Cancer Res..

[206] Miller D.C., Hafez K.S., Stewart A., Montie J.E., Wei J.T. (2003). Prostate carcinoma presentation, diagnosis, and staging: An update form the National Cancer Data Base. Cancer.

[207] Hagemann I.S. (2016). Molecular Testing in Breast Cancer: A Guide to Current Practices. Arch. Pathol. Lab. Med..

[208] Jang S., Atkins M.B. (2013). Which drug, and when, for patients with BRAF-mutant melanoma?. Lancet Oncol..

[209] Lindeman N.I., Cagle P.T., Beasley M.B., Chitale D.A., Dacic S., Giaccone G., Jenkins R.B., Kwiatkowski D.J., Saldivar J.S., Squire J. (2013). Molecular testing guideline for selection of lung cancer patients for EGFR and ALK tyrosine kinase inhibitors: Guideline from the College of American Pathologists, International Association for the Study of Lung Cancer, and Association for Molecular Pathology. J. Mol. Diagn..

[210] Georgi B., Korzeniewski N., Hadaschik B., Grullich C., Roth W., Sultmann H., Pahernik S., Hohenfellner M., Duensing S. (2014). Evolving therapeutic concepts in prostate cancer based on genome-wide analyses (review). Int. J. Oncol..

